# Effect of oral glutamine supplementation on growth and glutamine and glucose metabolism in suckling piglets

**DOI:** 10.1093/jas/skaf201

**Published:** 2025-06-18

**Authors:** Quentin L Sciascia, Daria De Leonardis, Solvig Görs, Andreas Vernunft, Anja Eggert, Jürgen Zentek, Cornelia C Metges

**Affiliations:** Research Institute for Farm Animal Biology (FBN), Nutrition and Metabolism, 18196 Dummerstorf, Germany; Research Institute for Farm Animal Biology (FBN), Nutrition and Metabolism, 18196 Dummerstorf, Germany; Research Institute for Farm Animal Biology (FBN), Nutrition and Metabolism, 18196 Dummerstorf, Germany; Research Institute for Farm Animal Biology (FBN), Nutrition and Metabolism, 18196 Dummerstorf, Germany; Research Institute for Farm Animal Biology (FBN), Nutrition and Metabolism, 18196 Dummerstorf, Germany; Department of Veterinary Medicine, Institute of Animal Nutrition, Freie Universität Berlin, 14195 Berlin, Germany; Research Institute for Farm Animal Biology (FBN), Nutrition and Metabolism, 18196 Dummerstorf, Germany

**Keywords:** Glutamine, glucose, low birthweight, oxidation, piglet, stable isotope tracers

## Abstract

The increase in litter size has led to a higher proportion of low birthweight (**LBW**) piglets, which are disadvantaged in terms of later growth and organ maturation. Supplementing with glutamine (Gln) has been proposed to improve this situation. We investigated the metabolism of Gln and glucose (Glc) in LBW and normal (**NBW**) birthweight (**BiW**) suckling piglets supplemented with Gln or water (W). Forty-six neonatal male German Landrace piglets with a BiW of 1.04 ± 0.02 kg for LBW piglets and 1.55 ± 0.02 kg for NBW piglets were assigned to one of four treatments in a 2 × 2 factorial design (LBW/NBW; Gln/W). Half of the groups received either 1 g/kg bodyweight (BW)/d of Gln (LBW-Gln, NBW-Gln; *n* = 12) dissolved in water, or water (W) as a control (LBW-W, NBW-W; *n* = 11), until age 15 d. At 12 d, piglets were implanted a jugular vein catheter and then underwent oral tests: on 14 d with ^13^C_5_-Gln/Gln, on 15 d with lactulose/mannitol (Lac/Man) and on 16 d with ^13^C_6_-Glc/Glc and xylose (Xyl). After a baseline blood sample, blood samples were collected over 5 h. Enrichments of ^13^C tracers in plasma and ^13^CO_2_ in red blood cells (**RBC**) were measured, along with plasma concentrations of Lac, Man, Xyl, Glc, lactate, urea, and amino acids. We calculated plasma Gln and Glc rate of appearance (Ra), ^13^C recovery (^13^C REC) in RBC CO_2_ from ^13^C_5_-Gln and ^13^C_6_-Glc as a proxy of whole-body oxidation and ^13^C_6_-Gln conversion to ^13^C_3_-Glc to obtain a measure of gluconeogenesis. The BW, average daily gain (**ADG**), abdominal circumference (**ABC**), crown-rump length (**CRL**), body mass index (**BMI**), and ponderal index were measured. The BW of LBW piglets supplemented with Gln was lower than of those supplemented with W at 12, 13, and 15 d (*P* < 0.05). The BW, ADG, ABC, CRL, and BMI remained lower in LBW than in NBW piglets (*P* < 0.01). Plasma urea was higher in the Gln groups (*P* < 0.05). The Xyl and Man absorption tests indicated a greater intestinal absorptive capacity/area in LBW piglets (*P* < 0.05). The ^13^C REC values of Gln and Glc were higher in LBW than in NBW piglets (*P* < 0.01), whereas ^13^C REC of Gln was higher than that of Glc independently of BiW and supplementation (*P* = 0.005). Plasma ^13^C_6_-Glc Ra was higher than that of ^13^C_5_-Gln Ra in all groups (*P* < 0.01). The ^13^C_5_-Gln to ^13^C_3_-Glc conversion did not differ among groups (*P* > 0.1). In conclusion, LBW and NBW piglets differ in Gln and Glc metabolism, but Gln supplementation did not show a consistent effect on the measured parameters.

## Teaser

Glutamine and glucose metabolism differs between low and normal birthweight piglets, but glutamine supplementation does not consistently affect glutamine or glucose metabolism of low birthweight piglets, compared with normal birthweight littermates.

## Graphical Abstract

**Graphical Abstract:**
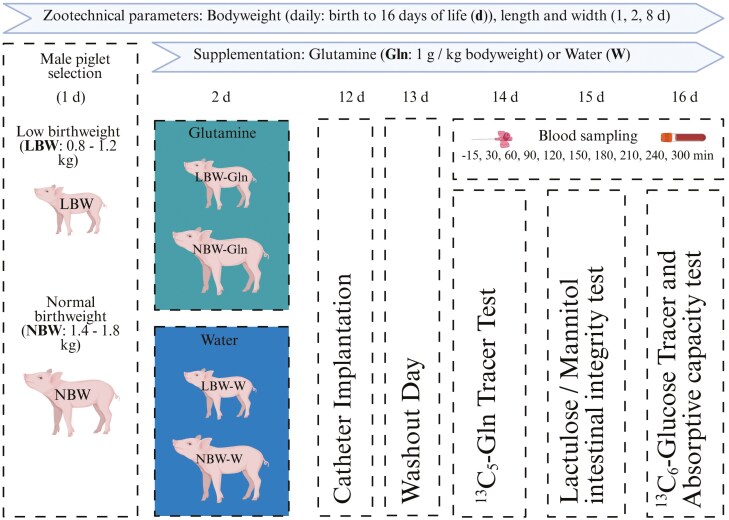

## Introduction

Increasing the number of weaned piglets per litter is a primary goal of modern pig farming systems (Bortolozzo et al., 2023). However, increasing litter sizes has resulted in a higher proportion of low birthweight (**LBW**) piglets being born, which have a higher preweaning morbidity and mortality (De Vos et al., 2014; Mugnier et al., 2022). The gastrointestinal tract is the main organ responsible for nutrient absorption, and studies have shown that gastrointestinal growth and function in suckling LBW piglets is impaired compared with normal birthweight (**NBW**) littermates (Michiels et al., 2013; De Vos et al., 2014; Tao et al., 2019). These impairments include reduced intestinal absorptive surface area and thus nutrient absorption and compromised gut barrier function, in particular in neonatal piglets with intrauterine growth restriction (**IUGR**), leading to increased susceptibility to infections when challenged by environmental pathogens (Everaert et al., 2017). Several nutritional interventions in suckling and postweaning pigs have been reported to improve intestinal growth and function in LBW pigs (Declerck et al., 2016; Blavi et al., 2021), of which one is glutamine (**Gln**).

The majority of in vivo Gln supplementation studies in pigs have been conducted during the postweaning period. These results show improved intestinal integrity, which limits the entrance of luminal pathogenic bacteria into the bloodstream, as well as improved intestinal structure—thus improving nutrient absorption and immune and antioxidative status, as evidenced by increased white blood cell counts and glutathione concentrations (Wang et al., 2009). The few Gln supplementation studies conducted in mixed birthweight and LBW suckling piglets report increased bodyweight (**BW**), average daily gain (**ADG**), and intestinal villus height and villus:crypt depth ratio, markers of improved intestinal nutrient absorption (Haynes et al., 2009; Wu et al., 2011; Yang et al., 2018; Li et al., 2022; Schregel et al., 2022). However, to date, it is only incompletely known if and how Gln improves intestinal growth and function in suckling piglets.

Glutamine is the primary fuel source for enterocytes, where the majority of dietary Gln is converted into glutamate (**Glu**) then alpha-ketoglutarate, which enters the Krebs cycle to serve as an oxidative fuel (Wu et al., 1994a; Janeczko et al., 2007; Cruzat et al., 2018). Arterio-venous difference studies in preweaning piglets have shown that Gln was the only amino acid (**AA**) extracted by the small intestine, but Glu, alanine (**Ala**), arginine (**Arg**), asparagine (**Asn**), and citrulline (**Cit**) were released from the small intestine (Wu et al., 1994a). In their pioneering studies on milk-replacer fed piglets, Stoll and Burrin (2006) reported that more than 95% of the dietary glutamine is utilized in intestinal tissues, but only 55% to 70% of Gln is oxidized to CO_2_. Additionally, alpha-ketoglutarate derived from Gln breakdown enters the Krebs cycle, where it is converted into oxaloacetate and subsequently removed to serve as a precursor for glucose production (Arnold and Finley, 2023) in the liver and kidney (Jungas et al., 1992), and differences in its production between LBW and NBW pigs may suggest differences in the ability to derive energy from Gln. In vitro studies using mouse and rat jejunum incubated with ^14^C-Gln showed that Gln reduces the oxidation of Glucose (**Glc**) carbon in the Krebs cycle (Kight and Fleming, 1995; Quan et al., 1998), indicating that Gln supplementation may impact the metabolism of Glc, the second most preferred fuel source of enterocytes (Wu et al., 1995).

Therefore, the hypotheses of this study were 1) that oral Gln supplementation improves the growth of LBW suckling piglets, 2) that improved growth is associated with changes in the in vivo metabolism of Gln and Glc (as measured with uniformly labeled ^13^C_5_-Glutamine [^13^C_5_-Gln] and ^13^C_6_-Glucose [^13^C_6_-Glc]), and 3) that in vivo intestinal barrier function and absorptive capacity are also improved .

## Material and Methods

### Ethics statement

All experimental procedures were approved by the State Office for Agriculture, Food Safety, and Fishery Mecklenburg-Western Pomerania, Germany (permission No. 7221.3-1-022/21) and were performed following the German Animal Welfare Act and the Directive 2010/63/EU for the Protection of Vertebrate Animals Used for Experimental and Other Scientific Purposes.

### Experimental animals and design

**Figure 1.**
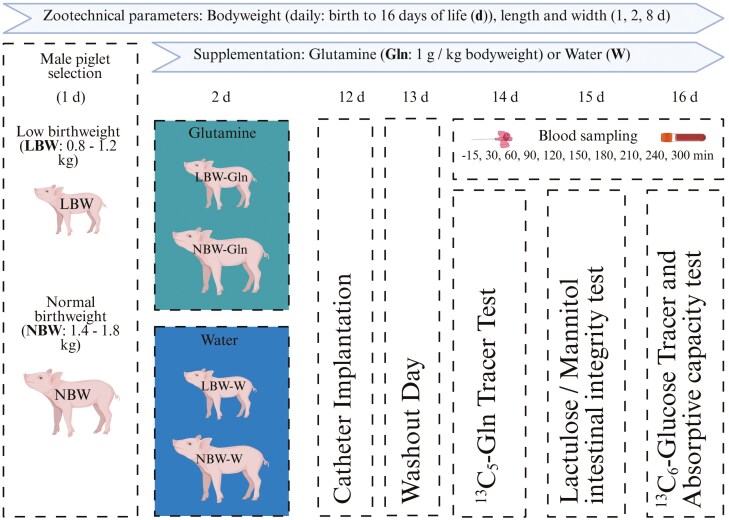
Scheme of experimental design showing metabolic studies in piglets with low and normal birthweight supplemented with glutamine (Gln) or water (W) starting at age 2 d^1^ (Created in BioRender). Birthweight range of male low (LBW) and normal birthweight (NBW) piglets selected were 0.8–1.2 and 1.4–1.8 kg, respectively. At birth (1 day of life (d)), LBW and NBW littermates were selected and the following day (2 d) randomly allocated to supplementation groups, resulting in 4 experimental groups (LBW-Gln, LBW-W, NBW-Gln, NBW-W). At 12 d, piglets were implanted with a jugular catheter to enable frequent blood sampling. At 14 d, a Gln metabolism study was performed using a ^13^C_5_-Gln tracer, at 15 d, an intestinal barrier function and absorptive capacity test was done using lactulose/mannitol administration, and xylose at 16 d, and at 16 d, a Glc metabolism study was conducted using a ^13^C_6_-Glc tracer.

The study was carried out at the experimental pig facility of the Research Institute for Farm Animal Biology (**FBN**). Piglets were sourced from German Landrace sows (parity 2 to 9), which were kept under standard conditions for housing, insemination, and farrowing (Rehfeldt et al., 2011). Sows were fed pregnancy (11.4 MJ of metabolizable energy [**ME**]/kg, 12.6% crude protein [**CP**], 3.8 % ether extract [**EE**], 9% fiber) and lactation diets (13.2 MJ of ME/kg, 16.5% CP, 6% EE, 5.3% fiber; Trede & v. Pein, Kiel, Germany). Only male piglets were selected to remove the effect of sex from the study, and we have previously reported that Gln supplementation improved BW of LBW male piglets (Li et al., 2022). The overall trial design is shown in Figure 1. At birth (1 day of life (d)), LBW (0.8 to 1.2 kg; represents the lowest birthweight quartile of piglets born at the FBN experimental pig facility) and NBW (1.4 to 1.8 kg; represents the middle 50th percentile of piglets born at the FBN experimental pig facility) littermate pairs were selected from 18 litters (mean litter size: 17 ± 0.4 piglets/litter). The criteria to select the piglets were based on findings from the FBN experimental pig facility, according to which piglets with a birthweight (**BiW**) of less than 1.18 kg had a lower survival rate than piglets with a BiW of >1.18 kg (Mbuthia et al., 2025). In total, 56 piglets were selected. During the study, five LBW and five NBW piglets each were removed due to illness, failure to grow for two consecutive days or jugular catheter malfunction. The remaining LBW piglets had a mean BiW of 1.04 ± 0.02 kg (*n* = 23), whilst NBW littermates had a mean BiW of 1.55 ± 0.02 kg (*n* = 23). We also examined our LBW-piglets for signs of IUGR (Amdi et al., 2013) but could not detect any. All experimental piglets stayed with their birth dam and litter, which was standardized to a litter size of 14 piglets within 24 h of farrowing.

Within 4 h after birth and at 2 and 8 d, abdominal circumference (**ABC**; Douglas et al., 2016), crown-rump length (**CRL**), body mass index (**BMI**), and ponderal index (**PI**; Amdi et al., 2013) were determined. Average daily gain during the entire study (1 to 16 d), prior to (1 to 12 d), and post- (13 to 16 d) catheter surgery was calculated. Bodyweight was recorded daily (1 to 16 d), between 06:30 and 07:00 h.

At 2 d of age, LBW and NBW littermates were assigned to the two supplementation groups where the piglets were orally administered either 1 g/kg BW/d of Gln (LBW-Gln, *n* = 12; NBW-Gln, *n* = 12) dissolved in water or water (**W**) as a control (LBW-W, *n* = 11; NBW-W, *n* = 11), and continued until the day before euthanasia (15 d). Glutamine dosage (33% of daily dose, 3 times per d), times of supplementation (07:00, 12:00, and 17:00 h) and volume of water (3 mL per dose) were determined based on a previous study (Li et al., 2022). The staff was not blinded to the supplementation group. At the end of the experiment, at 16 d of age, the piglets were euthanized with T61 (MSD Tiergesundheit, Unterschleißheim, Germany) after induction of general anesthesia with Azaperon (Stresnil, Elanco GmbH, Cuxhaven, Germany) and Ketamin (Ursotamin, Serumwerk, Bernburg, Germany).

### Catheter implantation and blood sampling

At 12 d, piglets were transferred to the surgery room of the FBNs experimental pig facility, where a jugular catheter was implanted (De Leonardis et al., 2023), to enable frequent blood sampling. Two different anesthesia protocols were used during catheter implantation. Initially, 18 piglets were anesthetized with azaperone/ketamine, followed by 28 piglets with isoflurane. The type of anesthesia was changed as it was observed that azaperone/ketamine led to a decrease in piglet BW due to a longer recovery time from the anesthesia (De Leonardis et al., 2023). The surgery wound and the exit point of the catheter were treated with an ointment containing iodine (Vet-Sept100 mg/g PVP iodine; Livisto, Hamburg, Germany) and covered by a 5 × 5 cm piece of a soft self-adhesive plaster (Fixmull stretch, 5 cm × 10 m, Hypafix, Hamburg, Germany). A first layer of soft cotton bandage (10 cm × 3 m, Cellona, Rengsdorf, Germany) was draped crosswise around the chest. A cohesive bandage (Petflex cohesive bandage, 5 cm, Covetrus, Hamburg, Germany) was used as a second layer for final coverage to protect the catheter from contamination or bites from littermates. On the day of the surgery, the piglets did not receive the 12:00 h Gln or water supplementation. After washing out the anesthetic on the 13th d, four oral tests were performed for which frequent blood samples were collected. At 14 d of age, a Gln metabolism study was performed using the stable isotope-labeled tracer ^13^C_5_-Gln; at 15 d, an intestinal barrier function/absorptive capacity test was done using lactulose/mannitol (**Lac/Man**) and xylose (**Xyl**) administration (16 d); and at 16 d, a Glc metabolism study was conducted using the stable isotope-labeled tracer ^13^C_6_-Glc (see below). One hour before the administration of the tracer bolus or test substrate (6:00 h), experimental piglets were separated from the sow, and a −15 min basal blood sample was taken (0.5 mL). After the tracer bolus or test substrate was administered, blood samples were taken at 30, 60, 90, 120, 150, 180, 210, 240, and 300 min (0.5 mL) and replaced with an equal volume of saline solution (0.9%). The piglets were returned to their litter between the blood sampling time points so that they could suckle. Blood was collected into EDTA tubes (Minicollect 0.5 mL K_3_EDTA, Greiner Bio-One GmbH, Frickenhausen, Germany) and immediately put on ice. Samples were centrifuged at 1573 × *g* and 4 °C for 20 min; plasma and red blood cells (**RBC**; only for the tracer tests) were harvested and stored at −80 °C, respectively, until analysis.

### Tracer studies with ^13^C_5_-glutamine and ^13^C_6_-glucose

At 14 d, a Gln metabolism study was performed by administering an oral bolus of Gln (0.33 g/kg BW) together with the tracer ^13^C_5_-Gln (10 mg/kg BW, 99.5 atom% ^13^C, Sigma-Aldrich, Darmstadt, Germany). At 16 d, after ^13^C abundance had been returned to the natural baseline, a Glc metabolism study was conducted by administering an oral bolus of ^13^C_6_-Glc (10 mg/kg BW, 99 atom% ^13^C, Cambridge Isotope Laboratories, Inc., Massachusetts, USA) together with Glc (0.4 g/kg BW, Roth, Karlsruhe, Germany). In both studies, the bolus dose was weighed into a disposable syringe and the tracer was suspended in water immediately before administration. To ensure the entire bolus was given, the syringe was filled with an additional volume of water and administered to the piglet.

### ^13^C enrichments of glutamine and glucose in plasma and carbon dioxide in red blood cells

To measure the ^13^C_5_-Gln enrichment in plasma samples collected after the Gln tracer administration (14 d), protein precipitation of 50 µL of plasma was performed with acetonitrile. The dried supernatants were reconstituted with 60 µL of ultrapure water, 35 µL of ethanol, and 10 µL pyridine, and 10 µL ethyl chloroformate was added to form glutamine-di-N-ethoxycarbonyl ethyl esters (Montigon et al., 2001). The samples were allowed to stand for 5 min at room temperature, then 500 µL of saturated NaHCO_3_ solution and 2 mL of dichloromethane were added and shaken. After a short centrifugation, 1.000 × *g* for 5 min, the aqueous phase (upper layer) was discarded and anhydrous Na_2_SO_4_ was added to dry the organic phase. The samples were transferred in a new tube and evaporated to dryness with N_2_ at 50 °C. Derivatives were redissolved in 100 µL of dichloromethane and analyzed by GC-MS (QP2010, coupled with GC 2010, Shimadzu, Duisburg, Germany) using a fused silica column (Ultra 2, 50 m length, 0.32 mm inner diameter, 0.52 µm film thickness, Agilent, Waldbronn, Germany). The gas chromatograph was operated with helium as carrier gas in the flow control mode with a linear velocity of 35.5 cm/s. A sample volume of 1 µL was transferred into the injector (AOC-20s, Shimadzu) at a split ratio of 5:1, while the injector and transfer line were held at 250 °C. The ion source was set at 200 °C and the detector at 1.2 kV. The temperature program was starting at 130 °C held for 1 min; 10 °C/min until 300 °C held for 5.5 min (Montigon et al., 2001).

The retention time of Gln was 13.5 min. The fragments m + 0, m + 1, m + 2, m + 3, and m + 4 (*m/z* 84 to *m/z* 88) were recorded in electron impact ionization mode and measured by selected-ion monitoring. The peak area ratios m + 4/m + 0 were converted to sample enrichments (molar percent excess; **MPE**) using a calibration curve based on standards with known enrichments of ^13^C_5_-Gln from 0 to 2.5 MPE.

The enrichment of plasma ^13^C_6_-Glc from samples collected after the Glc tracer administration (16 d) was analyzed in aldonitrile-pentaacetate derivative as described (Junghans et al., 2010) with the exception that 25 µL plasma was used and the derivative was dissolved in 100 µL of ethyl acetate instead of isooctane. The retention time of Glc was 6.2 min and the diagnostic fragments m + 0 and m + 6 were *m/z* 328 and *m/z* 334 (Junghans et al., 2010; Steinhoff-Wagner et al., 2011).

To estimate the rate of conversion of Gln to Glc carbon, as a measure of gluconeogenesis, 25 µL plasma samples (from ^13^C_5_-Gln administration at 14 d) was used to measure ^13^C_3_-Glc relative to the ^13^C_5_-Gln enrichment. The ^13^C_3_-Glc enrichment was determined as described (Junghans et al., 2010; Steinhoff-Wagner et al., 2011) with the derivatives dissolved in 100 µL of ethyl acetate. The diagnostic ions for ^13^C_3_-Glc were m + 0 (*m/z* 328) and m + 3 (*m/z* 331).

The ^13^C enrichment in RBC CO_2_ derived from tracer Gln and Glc oxidation was determined by gas isotope ratio mass spectrometry (DELTA plus XL, Thermo Quest Bremen, Germany) and the Gas Bench II (Finnigan, Bremen, Germany) (Junghans et al., 2007; Steinhoff-Wagner et al., 2011). The CO_2_ was isolated from 100 µL of RBC and incubated with 100 µL lactic acid in 4 mL glass tubes with septum screw caps (preflushed with argon). Red blood cells were used in this study instead of breath samples after results from a pretest (*n* = 4 piglets) showed that following administration of the Gln and Glc tracers as described above, the ^13^C enrichments in CO_2_ in RBC and breath samples were comparable (Supplementary Figure S1). The coefficient of determination between the ^13^C enrichment in CO_2_ of RBC and breath was *R*^2^ = 0.98 and *R*^2^ = 0.99 from ^13^C_5_-Gln and ^13^C_6_-Glc oxidation, respectively. In addition, RBC were a byproduct from blood samples following centrifugation to obtain plasma, and using the RBC removed additional stress as the piglets did not have to wear a face mask used for breath sample collection.

### Glutamine and glucose rate of appearance and oxidation, and glutamine conversion to glucose

To derive kinetic parameters for plasma ^13^C_5_-Gln, ^13^C_6_-Glc, ^13^C_3_-Glc, and RBC ^13^CO_2_, area under the enrichment-time-curves (**AUC**, mole or atom percent excess [MPE or APE] × min), maximal enrichment (***E***_**max**_, MPE or APE), and time to achieve *E*_max_ (***T***_**max**_, min) were determined by best peak curve fit (TableCurve 2D v5.01.01; Cranes Software International Ltd.).

The plasma rate of appearance (**Ra**) was calculated for Gln and Glc, using the following equation:

Ra [mmol/(kg×h)] = *D* / AUC (^13^C_5_-Gln or ^13^C_6_-Glc),

where *D* is the tracer dose (mmol/kg BW) and plasma AUC (^13^C_5_-Gln or ^13^C_6_-Glc) is the area under the enrichment-time-curve (MPE × h) for ^13^C_5_-Gln and ^13^C_6_-Glc, respectively (Gruse et al., 2015).

Synthesis of Glc from Gln carbon was estimated by the conversion of ^13^C_5_-Gln into ^13^C_3_-Glc and is presented as the quotient of plasma AUC (^13^C_3_-Glc) and plasma AUC (^13^C_5_-Gln) (%) reflecting gluconeogenesis.

The ^13^C recovery of total ^13^C (^**13**^**C REC**) (Metges and Wolfram, 1991) in RBC CO_2_ derived from the oxidation of ^13^C_5_-Gln and ^13^C_6_-Glc tracers and the ^13^C content of bolus Gln and Glc administered (relative to basal plasma ^13^C content) together with the tracers was used as a measure of Gln and Glc oxidation using the following equations:

*n* (^13^C in CO_2_) = AUC (^13^CO_2_) / 100% *× t × r* (CO_2_)

^13^C REC = *n* (^13^C in CO_2_) / *n* (^13^C in tracer dose) × 100%,

where *n* is the ^13^C amount (mmol), AUC (^13^CO_2_) is the area under the enrichment-time-curve (MPE × h) of RBC, and *t* is the sampling period of 5 h. The production of CO_2_ (*r* (CO_2_)) was determined earlier with 48 mmol/(kg × h) for suckling piglets at 21 d of age (*n* = 74, unpublished) using the doubly labeled water method. The ^13^C REC values were normalized with the RBC/breath ^13^CO_2_ correlation factors of 0.90 (^13^C_5_-Gln) and 0.98 (^13^C_6_-Glc), respectively, and expressed as % of ^13^C intake (Supplementary Figure S1).

### Free amino acid and metabolites in plasma

Plasma samples taken during the ^13^C_5_-Gln tracer test at 14 d were prepared to measure free amino acid concentration (**FAA**; Li et al., 2022). To investigate how Gln supplementation affected the AAs related to the Gln metabolic pathway, nine plasma FAA Ala, Arg, Asn, aspartate (**Asp**), Cit, Gln, Glu, proline (**Pro**), and serine (**Ser**) were quantified by HPLC (Schregel et al., 2022). Plasma concentrations of Glc, urea, and lactate were measured in 14 d and 16 d old piglets using a clinical chemistry analyzer (Das et al., 2016).

### Intestinal integrity, absorptive capacity, and malabsorption tests

Intestinal permeability, absorptive capacity, and malabsorption tests were carried out in suckling piglets at 15 and 16 d of age in response to Gln supplementation. One hour after isolation from the sow, the piglets were orally administered a bolus dose of Man (0.3 g/kg BW) and Lac (0.75 g/kg BW) freshly suspended in water (Huygelen et al., 2012). At 16 d, Xyl (0.4 g/kg BW, Roth, Karlsruhe, Germany) was orally administered together with Glc (see section: “Tracer studies with ^13^C_5_-glutamine and ^13^C_6_-glucose”). Xylose was used to test for malabsorption in the small intestine of LBW and NBW piglets, as Xyl is absorbed by a similar mechanism to Glc but is not fully metabolized (Huntley and Patience, 2018; Uken et al., 2021).

Blood samples were collected as described above to obtain plasma. Plasma Man and Lac concentrations were determined by HPLC with refractive index detection. After protein precipitation of 60 µL plasma with 120 µL 1.5 M HClO_4_, the mixture was neutralized with 60 µL of 2 M K_2_CO_3_ (Wu et al., 1999) followed by centrifugation at 50,000 × *g* and 4 °C for 20 min. The supernatant was diluted by factor 3 with ultrapure water. The separation of 10 µL of the diluted supernatant was performed on a Rezex RPM-Monosaccharide Pb^2+^ column 300 × 7.8 mm in combination with a Rezex ROA-OrganicAcid H^+^ (8%) Guard column 50 × 7.8 mm and a 4 × 3 mm Carbo-H pre-column (all Phenomenex, Aschaffenburg, Germany) at a flow rate of 0.6 mL/min at 75 °C with ultrapure water as eluent. An external multipoint calibration was performed and the limits of quantification in plasma were 0.03 mmol/L for lactulose and 0.06 mmol/L for mannitol, respectively.

Using the plasma collected at 16 d, Xyl concentration was also measured. After deproteinization of 60 µL of plasma with 40 µL of 1.5 M HClO_4_ and neutralization with 20 µL of 2 M K_2_CO_3_ (Wu et al., 1999), the mixture was centrifuged at 50,000 × *g* and 4 °C for 20 min. Plasma Xyl concentration was analyzed by HPLC (Junghans et al., 2010). The limit of quantification for xylose in plasma was 0.01 mmol/L, AUC, *E*_max_, and *T*_max_ were calculated as described above.

### Statistical analysis

The required number of animals was determined based on assumed effect sizes for the two main effects in a two-factorial experimental design: Cohen’s *f* = 0.5 for birthweight and *f* = 0.45 for supplementation. These effect sizes were derived from data reported by Li et al. (2022). Assuming a Type I error rate of 0.05 and a statistical power of 0.80, analysis indicated that a minimum of 11 animals per group was required to detect the specified effects. Sample size calculations were conducted in R using the ss.2way() function from the pwr2 package (Lu et al., 2017).

Three models were used to evaluate the data in this study. Model 1 was used to evaluate total and pre- and postsurgery ADG, AUC, *E*_max_, *T*_max_, ^13^C recovery, and Ra with the Generalized Linear Mixed Model (GLIMMIX) procedure from SAS (Version 9.4; SAS Institute Incorporated, Cary, North Carolina, USA) using a Gaussian model (model statement: distribution = Gaussian, link = Identity). The model included the fixed effects BiW class (LBW, NBW), Supplementation (Gln, W), and the interaction BiW × Supplementation, as well as Sow and Anesthetic (azaperone/ketamine, isoflurane) as random effects. The SLICE statement was used to perform partitioned analyses of the least-squares means for the BiW × Supplementation interaction. Model 2, used for the evaluation of BW, was the same as Model 1 but included the fixed effect of Age with repeated measurements on the same animal taken into account using the SUBJECT = animal option to define the blocks of the block-diagonal residual covariance matrix and the TYPE = AR (1) option to define their covariance structure. The SLICE statement was used to perform partitioned analyses of the least-squares means for the BiW × Supplementation × Age interaction. The zootechnical parameters (ABC, CRL, BMI, BW, and PI), the plasma metabolite and FAA concentrations, the plasma ^13^C_5_-Gln and ^13^C_6_-Glc tracer enrichments, the plasma ^13^C_3_-Glc and RBC ^13^CO_2_ enrichments, and the concentration of plasma mannitol and Xyl were assessed by model 3. Model 3 was the same as Model 2, with repeated measurements (Age or Time) on the same animal taken into account using the SUBJECT = animal option to define the blocks of the block-diagonal residual covariance matrix and the TYPE = CS option to define their covariance structure.

Model selection was based on Akaike’s information criterion (Ludden et al., 1994). Sow was defined as a random factor, which allowed modeling of littermates from the same sow and inference about the fixed effects; piglet was the experimental unit. Data were tested for outliers within the interaction BiW × Supplementation or BiW × Supplementation × Age or Time using sgsplot (SAS). Outliers were defined as 1.5 times the interquartile range greater than the third quartile or 1.5 times the interquartile range less than the first quartile. Identified outliers were only removed if they prevented data being normally distributed. Datasets normality of the residuals was tested using the Shapiro–Wilks test (SAS), and when data met the assumptions of normal distribution, differences were assessed using the Tukey–Kramer test. Differences were considered significant at *P* ≤ 0.05.

## Results

### Zootechnical parameters

**Figure 2.**
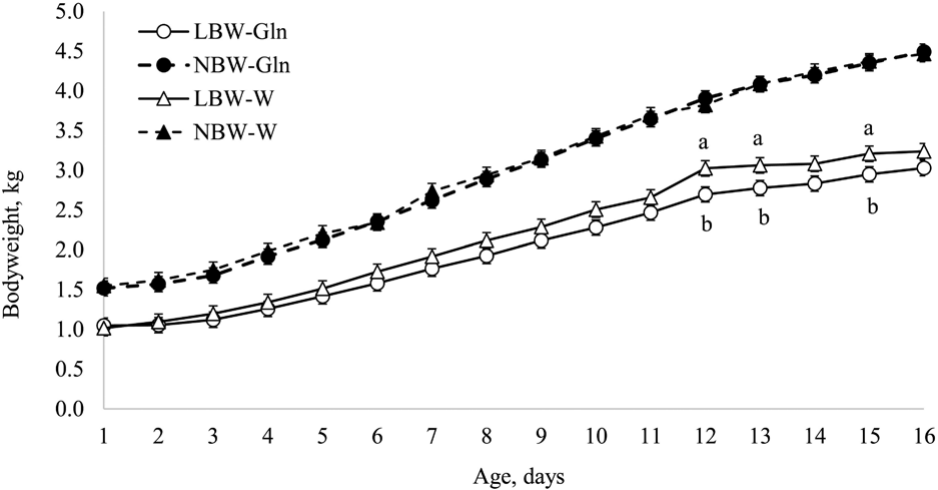
Increase in bodyweight during the experimental period in piglets with low and normal birthweight supplemented with glutamine (Gln; 1 g/kg bodyweight per d) or water starting at age 2 d.

Bodyweight was affected by BiW, Age, and the interaction BiW × Supplementation × Age (*P *< 0.001). At 12 (*P* = 0.01), 13 (*P* = 0.03), and 15 d (*P* = 0.05), BW of LBW-Gln was lower compared with LBW-W piglets (Figure 2). Within supplementation group, the BW of LBW piglets was lower than that of the NBW piglets during the entire experimental period (1 to 16 d), (*P *< 0.001; Figure 2).

The ABC, CRL, BMI, ADG, pre- (*P *< 0.001), and postsurgery ADG (*P* = 0.002) were affected by BiW, while ABC, CRL, and BMI (*P *< 0.001) were affected by Age, ABC was affected by Supplementation (*P* = 0.003), and ADG was affected by the interaction BiW × Supplementation (*P* = 0.02; Supplementary Table S1). Within each supplementation group, LBW piglets had lower ABC and CRL than NBW piglets (1, 2, and 8 d; *P *< 0.001; Supplementary Table S1) and at 8 d, ABC was lower in NBW-Gln compared with NBW-W (*P* = 0.03), and LBW-Gln to LBW-W (*P* = 0.002; Supplementary Table S1). At 1 and 2 d, LBW-Gln piglets had a lower BMI than NBW-Gln (*P* < 0.005), and at 2 and 8 d, the BMI of LBW-W piglets was lower than that of NBW-W (*P* ≤ 0.01) (Supplementary Table S1). Within each supplementation group, ADG was higher in NBW compared with LBW (*P *< 0.001), while presurgery ADG was lower in LBW-Gln piglets compared with NBW-Gln (*P *< 0.001), and postsurgery ADG was lower in LBW-W than NBW-W (*P* = 0.01; Supplementary Table S1).

### Glutamine tracer study

**Figure 3.**
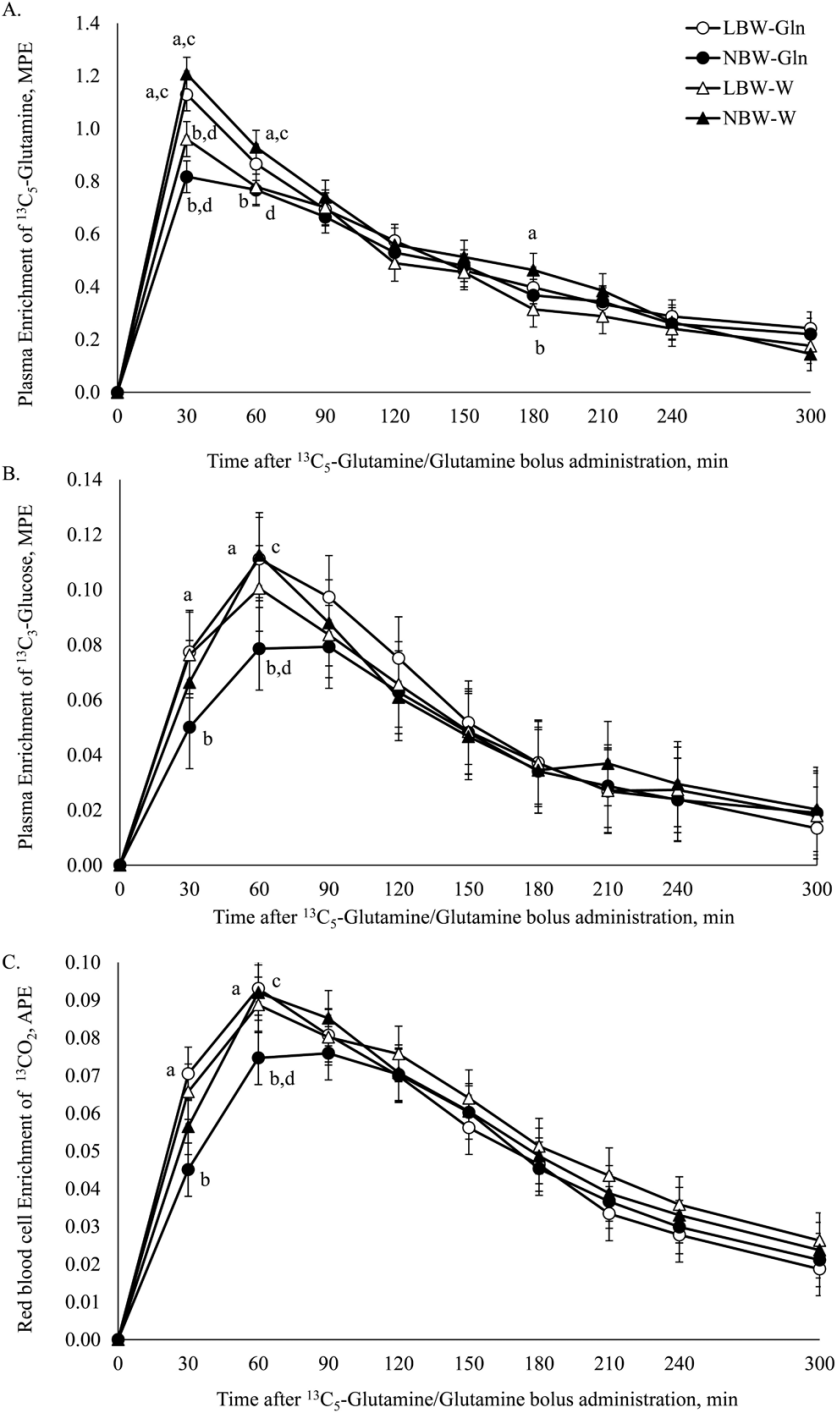
Course of ^13^C enrichment after administration of an oral bolus dose of glutamine (Gln; 0.33 g/kg bodyweight [BW]) plus ^13^C_5_-Gln (10 mg/kg BW) at 14 d of age, in low and normal birthweight male piglets supplemented with Gln (1 g/kg BW per d) or water (W), from 2 to 15 d of age. (A) Plasma enrichment of ^13^C_5_-Gln, MPE; (B) Plasma enrichment of ^13^C_3_-Glc synthesized from ^13^C_5_-Gln, MPE. (C) Red blood cell ^13^CO_2_ enrichment, APE.

Plasma enrichment of ^13^C_5_-Gln was affected by Time and the interaction BiW × Supplementation × Time. At 30 min post ^13^C_5_-Gln/Gln bolus, plasma ^13^C_5_-Gln enrichment was higher in LBW-Gln compared with LBW-W (*P* < 0.001; Figure 3A) and NBW-Gln (*P* = 0.05) piglets, and higher in NBW-W than NBW-Gln (*P *< 0.001) and LBW-W piglets (*P* = 0.001; Figure 3A). At 60 min postbolus, plasma ^13^C_5_-Gln enrichment was higher in NBW-W than NBW-Gln (*P* = 0.04) and LBW-W piglets (*P* = 0.04; Figure 3A). At 180 min postbolus, plasma ^13^C_5_-Gln enrichment was lower in LBW-W than in NBW-W piglets (*P* = 0.05; Fig 3A).

Plasma ^13^C_3_-Glc enrichment derived from ^13^C_5_-Gln (Glc synthesized from Gln carbon) was affected by Time (*P *< 0.001). At 30 min, the plasma ^13^C_3_-Glc enrichment was higher in LBW-Gln than NBW-Gln piglets (*P* = 0.01; Figure 3B). At 60 min, plasma ^13^C_3_-Glc enrichment was lower in NBW-Gln than LBW-Gln (*P* = 0.01) and NBW-W (*P* = 0.01; Figure 3B).

The ^13^C enrichment of CO_2_ from RBC was affected by time (*P *< 0.001). At 30 and 60 min, LBW-Gln piglets had a higher RBC ^13^CO_2_ enrichment compared with NBW-Gln piglets (*P *< 0.01). At 60 min, ^13^CO_2_ enrichment in NBW-Gln was lower compared with NBW-W piglets (*P* = 0.03; Figure 3C).

Plasma ^13^C_5_-Gln *E*_max_ (*P* < 0.001), *T*_max_ (*P* = 0.04), and AUC (*P* = 0.04) were affected by the interaction of BiW × Supplementation (Table 1). Plasma ^13^C_5_-Gln *E*_max_ was higher in LBW-Gln compared with LBW-W (*P* = 0.05) and NBW-Gln piglets (*P* = 0.003), and lower in NBW-Gln than NBW-W (*P* = 0.03; Table 1). The *T*_max_ of RBC ^13^CO_2_ was affected by the interaction of BiW × Supplementation (*P* = 0.008). The *T*_max_ of RBC ^13^CO_2_ was longer in NBW-Gln than LBW-Gln (*P* = 0.02) and NBW-W (*P* = 0.04) piglets. The plasma ^13^C_3_-Glc *T*_max_ was affected by the interaction of BiW × Supplementation (*P* = 0.004). The ^13^C_3_-Glc *T*_max_ was longer in NBW-Gln than LBW-Gln (*P* = 0.001) and NBW-W piglets (*P* = 0.01; Table 1). The % conversion of ^13^C_5_-Gln to ^13^C_3_-Glc (*P* = 0.05) was affected by the interaction of BiW × Supplementation but did not differ among experimental groups (Table 1). The ^13^C_5_-Gln Ra in plasma (*P* = 0.04; Table 2) was affected by the interaction of BiW × Supplementation and Gln ^13^C REC was affected by BiW (*P* < 0.001; Table 2). The ^13^C REC was higher in LBW-Gln than NBW-Gln (*P* = 0.001) and LBW-W than NBW-W littermates (*P* = 0.01; Table 2).

**Table 1.** The *E*_max_, *T*_max_, AUC, and % conversion determined after an oral bolus dose of glutamine (Gln; 0.33 g/kg bodyweight [BW]) plus ^13^C_5_-Gln (10 mg/kg BW) at 14 d of age, in low (LBW) and normal (NBW) birthweight (BiW) male piglets supplemented with Gln (1 g/kg BW per d) or water (W), from 2 to 15 d of age

Parameters^1^^,^^2^	Gln				Water				*P*-value^3^		
	LBW	SEM	NBW	SEM	LBW	SEM	NBW	SEM	BiW	Suppl	Interaction
*E*_max_											
Plasma ^13^C_5_-Gln, MPE	1.18^a,c^	0.08	0.89^b,d^	0.07	0.96^d^	0.08	1.13^c^	0.09	0.37	0.93	0.001
RBC ^13^CO_2_-Gln, APE	0.10	0.01	0.08	0.01	0.09	0.01	0.09	0.01	0.22	0.68	0.28
Plasma ^13^C_3_-Glc, MPE	0.12	0.02	0.09	0.02	0.10	0.02	0.11	0.02	0.26	0.93	0.19
*T*_max_, min											
Plasma ^13^C_5_-Gln	31.3	3.96	37.8	3.68	36.6	4.10	29.1	4.29	0.87	0.70	0.04
RBC ^13^CO_2_-Gln	59.1^b^	5.67	80.0^a,c^	5.43	74.9	5.67	61.1^d^	6.65	0.55	0.79	0.01
Plasma ^13^C_3-_Glc	56.8^b^	4.80	75.1^a,c^	4.50	61.6	5.01	56.5^d^	5.20	0.08	0.20	0.004
AUC											
Plasma ^13^C_5_-Gln, MPE × min	164	13.6	142	12.8	138	14.2	159	14.7	0.97	0.78	0.04
RBC ^13^CO_2_-Gln, APE × min	15.4	1.21	14.2	1.18	16.1	1.24	14.5	1.34	0.12	0.70	0.83
Plasma ^13^C_3_-Glc, MPE × min	15.1	3.23	12.8	3.15	14.3	3.25	13.0	3.32	0.32	0.88	0.79
Conversion, %											
^ 13^C_5_-Gln to ^13^C_3_-Glc	8.24	1.35	9.78	1.24	10.4	1.34	7.37	1.39	0.49	0.93	0.05

^1^Values are least-square means ± SEM, LBW-Gln; *n* = 9–11, NBW-Gln; *n* = 10–12, LBW-W; *n* = 10–11; NBW-W, *n* = 8–9 animals per group.

^2^Abbreviations: AUC, area under the curve; *E*_max_, maximum enrichment; RBC, red blood cells; Suppl, Supplementation; *T*_max_, time to maximum enrichment; Glc, glucose.

^3^GLIMMIX F test: Interaction is BiW × Suppl.

^a,b^Different from NBW piglets within Suppl group (*P* ≤ 0.05).

^c,d^Different from W supplemented piglets within BiW group (*P* ≤ 0.05).

**Table 2.** The rate of appearance and ^13^C recovery of glutamine and glucose, determined after an oral bolus containing glutamine (Gln, 0.33 g/kg bodyweight [BW]) plus ^13^C_5_-Gln (10 mg/kg BW) at 14 days of age, and glucose (Glc, 0.4 g/kg BW) plus ^13^C_6_-Glc (10 mg/kg BW) at 16 d of age, respectively, in low (LBW) and normal (NBW) birthweight (BiW) male piglets supplemented with Gln (1 g/kg BW per d) or water (W), from 2 to 15 d of age

Parameters^1^^,^^2^	Gln				Water				*P*-value^3^		
	LBW	SEM	NBW	SEM	LBW	SEM	NBW	SEM	BiW	Suppl	Interaction
Rate of appearance, mmol/(kg × h)											
Plasma ^13^C_5_-Gln	2.68^f^	0.25	3.11^f^	0.24	3.07^f^	0.26	2.62^f^	0.28	0.96	0.85	0.04
Plasma ^13^C_6_-Glc	5.63^e^	0.65	5.48^e^	0.59	5.07^e^	0.65	6.61^e^	0.65	0.28	0.67	0.19
^13^C Recovery, %											
^ 13^C_5_-Gln	69.7^a^	8.63	45.9^b^	8.63	71.5^a^	8.83	51.1^b,e^	8.83	0.00	0.50	0.72
^ 13^C_6_-Glc	54.8^a^	4.42	39.0^b^	4.61	53.1^a^	4.89	27.1^b,f^	5.16	0.00	0.18	0.25

^1^Values are least-square means ± SEM, LBW-Gln; *n* = 8–11, NBW-Gln; *n* = 10–12, LBW-W; *n* = 9–11; NBW-W, *n* = 8–9 animals per group.

^2^Abbreviations: RBC, red blood cells; Suppl, Supplementation.

^3^GLIMMIX F test (comparison between each experimental group, within each test): Interaction is BiW × Suppl.

^a,b^Different from NBW piglets within Suppl group (*P* ≤ 0.05).

^c,d^Different from W supplemented piglets within BiW group (*P* ≤ 0.05).

^e,f^Different from piglets given a bolus of Gln (0.33 g/kg BW) plus ^13^C_5_-Gln (10 mg/kg BW) (*P* ≤ 0.05).

### Glucose tracer study

**Figure 4.**
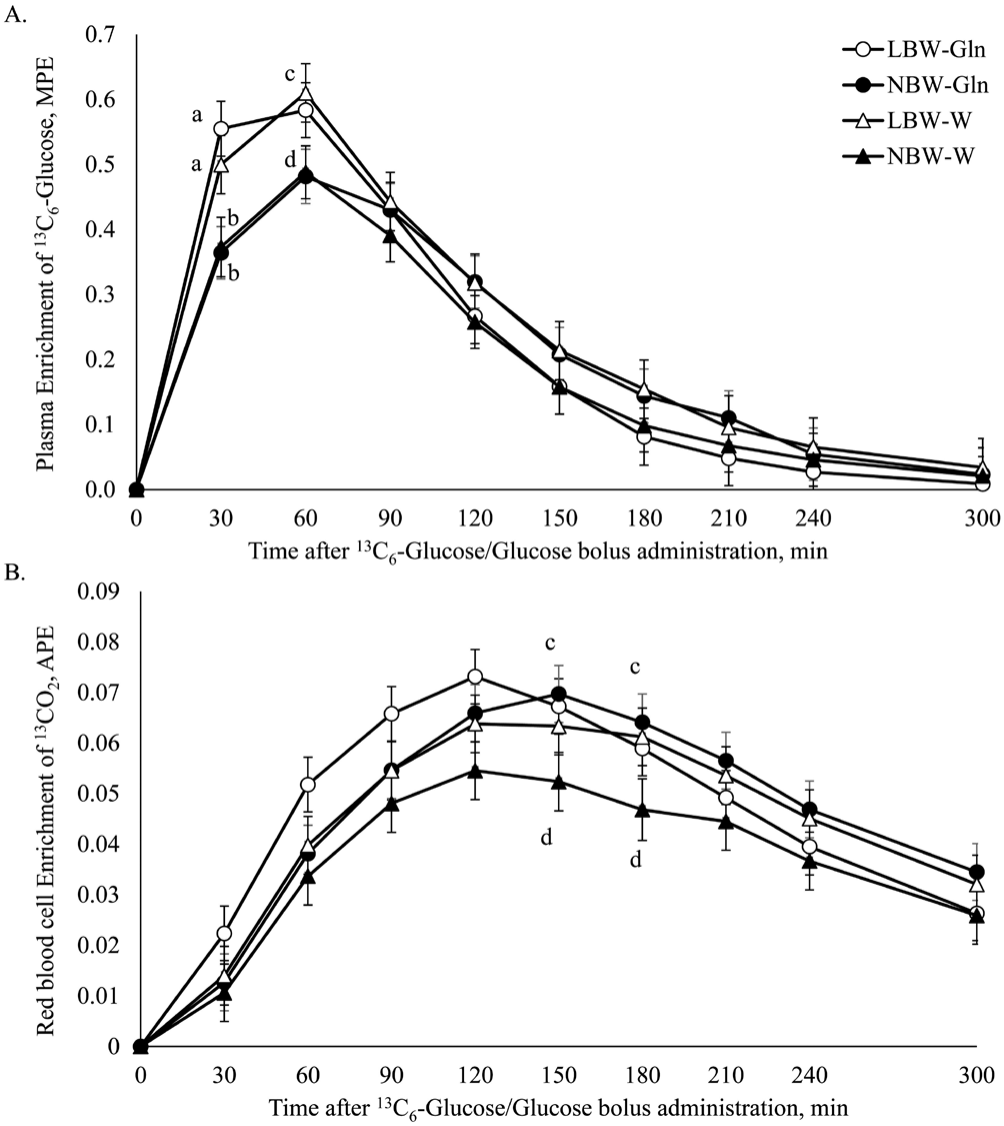
Course of ^13^C enrichment after administration of an oral bolus dose of glucose (Glc; 0.4 g/kg bodyweight [BW]) plus ^13^C_6_-Glc (10 mg/kg BW) at 16 d of age, in low and normal birthweight male piglets supplemented with glutamine (Gln; 1 g/kg BW per d) or water (W), from 2 to 15 d of age. (A) Plasma enrichment of ^13^C_6_-Glc, MPE; (B) Red blood cell ^13^CO_2_ enrichment, APE.

The plasma enrichment of ^13^C_6_-Glc was affected by Time (*P *< 0.001) and the interaction BiW × Time (*P* < 0.001; Figure 4A). At 30 min, ^13^C_6_-Glc enrichment of LBW piglets was higher than that of NBW irrespective of supplementation (*P *< 0.001); while at 30 and 60 min (*P* ≤ 0.04), plasma ^13^C_6_-Glc enrichment was higher in the LBW-W compared with NBW-W group (Figure 4A). The ^13^C enrichment in RBC CO_2_ was affected by Time (*P *< 0.001) and the interaction BiW × Supplementation × Time (*P* = 0.01). At 150 and 180 (*P* = 0.03) min, ^13^C enrichment in CO_2_ of RBC was higher in NBW-Gln compared with NBW-W piglets (Figure 4B).

The ^13^C enrichment in RBC CO_2_*E*_max_ was affected by Supplementation (*P* = 0.04) and *T*_max_ was affected by the interaction between BiW × Supplementation (*P* = 0.04; Table 3). The *E*_max_ (*P* = 0.04) and AUC (*P* = 0.05) were higher in NBW-Gln than NBW-W, while *T*_max_ was longer in NBW-Gln than LBW-Gln (*P* = 0.02) and NBW-W (*P* = 0.05; Table 3). The ^13^C REC was affected by BiW (*P* < 0.001; Table 2). The Glc ^13^C REC was higher in LBW-Gln than NBW-Gln (*P* = 0.01) and LBW-W than NBW-W littermates (*P* < 0.001; Table 2).

**Table 3.** The *E*_max_, *T*_max_, and AUC determined after an oral bolus dose of glucose (Glc; 0.4 g/kg bodyweight [BW]) plus ^13^C_6_-Glc (10 mg/kg BW) at 16 d of age, in low (LBW) and normal (NBW) birthweight (BiW) male piglets supplemented with glutamine (Gln; 1 g/kg BW per d) or water (W), from 2 to 15 d of age

Parameters^1^^,^^2^	Gln				Water				*P*-value^3^		
	LBW	SEM	NBW	SEM	LBW	SEM	NBW	SEM	BiW	Suppl	Interaction
*E*_max_											
Plasma ^13^C_6_-Glc, MPE	0.68	0.07	0.52	0.06	0.61	0.07	0.51	0.07	0.06	0.64	0.66
RBC ^13^CO_2_-Glc, APE	0.07	0.01	0.07^c^	0.01	0.06	0.01	0.05^d^	0.01	0.12	0.04	0.38
*T*_max_, min											
Plasma ^13^C_6_-Glc	49.6	5.57	58.2	5.08	50.3	5.60	50.2	5.60	0.43	0.49	0.41
RBC ^13^CO_2_-Glc	116^b^	8.25	144^a,c^	8.26	125	8.37	120^d^	9.20	0.15	0.37	0.04
AUC											
Plasma ^13^C_6_-Glc, MPE × min	65.6	7.04	65.0	6.42	68.9	7.24	52.4	7.24	0.17	0.54	0.20
RBC ^13^CO_2_-Glc, APE × min	13.7	1.31	13.8^c^	1.31	12.7	1.34	10.1^d^	1.47	0.25	0.10	0.23

^1^Values are least-square means ± SEM, LBW-Gln; *n* = 8–11, NBW-Gln; *n* = 10–12, LBW-W; *n* = 9–11; NBW-W, *n* = 9 animals per group.

^2^Abbreviations: AUC, area under the curve; *E*_max_, maximum enrichment; RBC, red blood cells; Suppl, Supplementation; *T*_max_, time to maximum enrichment.

^3^GLIMMIX F test: Interaction is BiW × Suppl.

^a,b^Different from NBW piglets within Suppl group (*P* ≤ 0.05).

^c,d^Different from W supplemented piglets within BiW group (*P* ≤ 0.05).

Comparison of plasma Glc and Gln Ra showed that ^13^C_6_-Glc Ra [5.45 ± 0.35 mmol/(kg × h)] was higher than ^13^C_5_-Gln Ra [2.57 ± 0.35 mmol/(kg × h)] in all experimental groups (LBW-Gln and NBW-W, *P *< 0.001; NBW-Gln, *P* = 0.001; LBW-W, *P* = 0.01; Table 2). In contrast, ^13^C REC in RBCs as a proxy of oxidation was lower for ^13^C_6_-Glc than for ^13^C_5_-Gln (43.40 ± 4.17% vs. 52.80 ± 4.09%; *P* = 0.005). In the individual groups, only in NBW-W piglets, the ^13^C_6_-Glc ^13^C REC was lower than that of ^13^C_5_-Gln ^13^C REC (Table 2).

### Plasma concentrations of AAs during the glutamine tracer study

**Figure 5.**
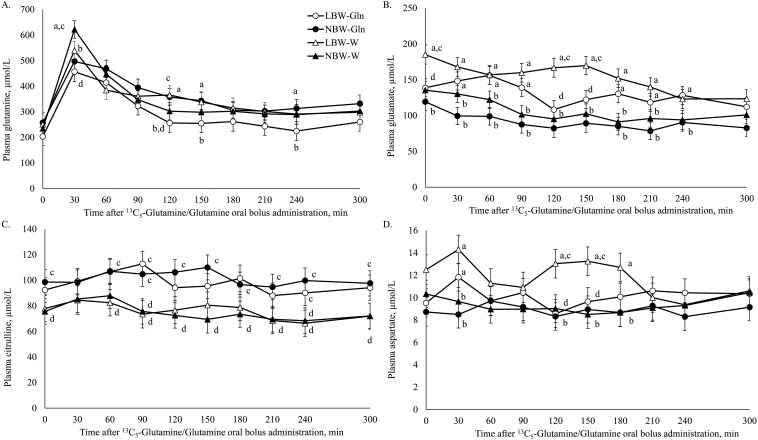
Plasma concentrations of (A) glutamine, (B) glutamate, (C) citrulline, and (D) aspartate prior to and after administration of an oral bolus dose of glutamine (Gln; 0.33 g/kg bodyweight [BW]) plus ^13^C_5_-Gln (10 mg/kg BW) at 14 d of age, in low and normal birthweight male piglets supplemented with Gln (1 g/kg BW per d) or water (W), from 2 to 15 d of age.

The plasma concentration of Gln was affected by BiW (*P* = 0.04) and Time (*P *< 0.001). At 30 min post ^13^C_5_-Gln/Gln bolus, the plasma Gln concentration was higher in NBW-W than NBW-Gln (*P* = 0.003) and LBW-W (*P* = 0.04; Figure 5A). At 120 min post ^13^C_5_-Gln/Gln bolus, the plasma Gln concentration was lower in LBW-Gln compared with LBW-W (*P* = 0.02) and NBW-Gln (*P* = 0.01). At 150 min (*P* = 0.03) and at 240 min (*P* = 0.04), Gln concentration was lower in LBW-Gln compared with NBW-Gln (Figure 5A).

Glutamate concentration in plasma was affected by BiW, Time (*P* < 0.001) and the interaction of BiW × Supplementation × Time (*P* = 0.03). Prior to the bolus being administered (−15 min), plasma Glu concentrations were higher in LBW-W than LBW-Gln (*P* = 0.01) and NBW-W (*P* = 0.004; Figure 5B). Plasma Glu concentration was higher in LBW-Gln compared with NBW-Gln between 30 and 90 min (*P* < 0.01), and 180 and 240 min (*P* ≤ 0.02). From 30 to 210 min (*P* ≤ 0.02), plasma Glu concentrations were also higher in LBW-W compared with NBW-W. At 120 and 150 min (*P* ≤ 0.01), plasma Glu concentrations were lower in LBW-Gln than LBW-W (Figure 5B).

Plasma Cit concentration was affected by Time (*P *< 0.001) and Supplementation (*P* = 0.004). Citrulline was higher in LBW-Gln than LBW-W piglets at 60, 90, and 240 min (*P* = 0.04), and in NBW-Gln compared with NBW-W at −15 min (*P* = 0.05), and at 90 to 240 (*P *< 0.05) min and 300 min (*P* = 0.03; Figure 5C).

Plasma Asp concentration was affected by BiW (*P* = 0.003) and the interactions Supplementation × Time (*P* = 0.02) and BiW × Supplementation × Time (*P* = 0.02). At 30 min, the concentration of plasma Asp was higher in LBW-Gln compared with NBW-Gln (*P* = 0.01), and in LBW-W compared with NBW-W (*P *< 0.001; Figure 5D). At 120 and 150 min, Asp concentrations were higher in LBW-W than LBW-Gln (*P* ≤ 0.02) and NBW-W piglets (*P* ≤ 0.001). At 180 min, the Asp concentration was higher in LBW-W than NBW-W (*P* = 0.001; Figure 5D).

Arginine concentration was higher at 60 min in NBW-W compared with LBW-W (102 vs. 128 μmol/L; *P* = 0.01), and in NBW-Gln compared with NBW-W (38.3 vs. 29.7 μmol/L; *P* = 0.04). The average concentration across the experimental period of plasma Ala, Arg, Asn, Pro, and Ser was not found to be different (Supplementary Table S2).

### Plasma metabolite concentrations during the glutamine and glucose tracer studies

At age 14 d, following administration of the ^13^C_5_-Gln/Gln bolus, plasma concentrations of Glc (*P* = 0.01) and lactate (*P *< 0.001) were affected by Time, while urea was affected by Time (*P *< 0.001), BiW (*P* = 0.02), and the interaction of Supplementation × Time (*P *< 0.001: Supplementary Table S3). Prior to ^13^C_5_-Gln/Gln bolus administration (−15 min), plasma Glc concentrations were higher in LBW-W compared with NBW-W and LBW-Gln piglets (*P* = 0.04; Supplementary Table S3). At 30 min, plasma urea was lower in NBW-W compared with LBW-W (*P *= 0.05) and NBW-Gln (*P* = 0.03; Supplementary Table S3).

At age 16 d, after administration of a ^13^C_6_-Glc/Glc bolus, the plasma concentration of Glc was affected by Time (*P* = 0.02), plasma lactate by Time, and the interaction of Supplementation × Time (*P *< 0.001), while urea was affected by Time (*P *< 0.001) and Supplementation (*P* = 0.01; Supplementary Table S4). Prior to bolus administration (−15 min), the concentration of plasma lactate was higher in LBW-Gln compared with NBW-Gln (*P *< 0.001) and LBW-W (*P *< 0.001). The plasma urea concentration was higher in NBW-Gln than NBW-W from 30 to 300 min (*P* < 0.05; Supplementary Table S4).

### Intestinal barrier integrity, absorptive capacity, and malabsorption tests

At 15 d of age, intestinal barrier function was determined using the Lac/Man test. The Man concentration in plasma was affected by Time (*P *< 0.001). At 150 min, the plasma mannitol concentration was higher in LBW-Gln compared with NBW-Gln (*P* = 0.04; Supplementary Table S5). The plasma Lac concentration was at or below the limit of quantification (data not presented).

**Figure 6.**
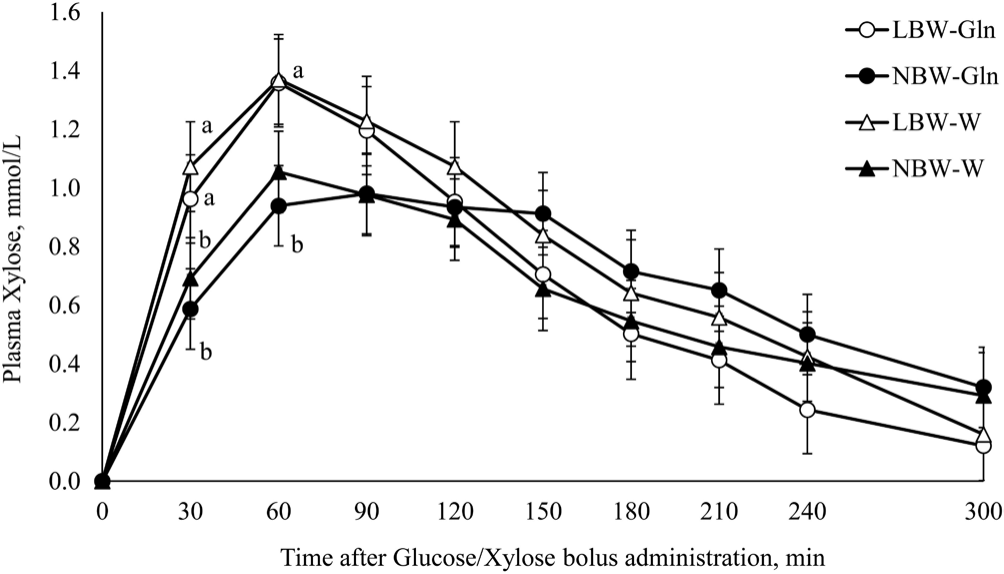
Plasma xylose concentrations prior to and after an oral bolus dose of glucose (0.4 g/kg bodyweight [BW]) and xylose (0.4 g/kg BW) at 16 d of age, in low and normal birthweight male piglets supplemented with glutamine (Gln; 1 g/kg BW per d) or water (W), from 2 to 15 d of age.

At 16 d, an intestinal absorptive capacity test was performed using Glc/Xyl. The plasma Xyl concentrations were affected by Time and the interaction of BiW × Time (*P *< 0.001). At 30 min postbolus, the plasma concentration of Xyl was higher in LBW piglets compared with their NBW littermates (*P* = 0.04), while at 60 min, the plasma concentration of Xyl was higher in LBW-Gln than NBW-Gln piglets (*P* = 0.02; Figure 6).

The plasma Xyl *T*_max_ was affected by BiW (*P* = 0.01). The *T*_max_ was longer in NBW-Gln than LBW-Gln (*P* = 0.004) and NBW-W groups (*P* = 0.05; Table 4).

**Table 4.** The *E*_max_, *T*_max_, and AUC of plasma xylose determined after an oral bolus dose of glucose (0.4 g/kg bodyweight [BW]) and xylose (0.4 g/kg BW) at 16 d of age, in low (LBW) and normal (NBW) birthweight (BiW) male piglets supplemented with glutamine (Gln; 1 g/kg BW per d) or water (W), from 2 to 15 d of age

Parameters^1^^,^^2^	Gln				Water				*P*-value^3^		
	LBW	SEM	NBW	SEM	LBW	SEM	NBW	SEM	BiW	Suppl	Interaction
*E*_max_, mM	1.35	0.27	1.27	0.26	1.21	0.30	0.92	0.30	0.24	0.20	0.49
*T*_max_, min	60.6^b^	10.1	101^a,c^	9.69	60.3	12.2	72.8^d^	9.84	0.01	0.21	0.15
AUC, mM × min	214	45.8	200	45.8	164	48.5	160	47.5	0.63	0.07	0.79

^1^Values are least-square means ± SEM, LBW-Gln; *n* = 8–9, NBW-Gln; *n* = 10–11, LBW-W; *n* = 5–9; NBW-W, *n* = 9–11 animals per group.

^2^Abbreviations: AUC, area under the curve; *E*_max_, maximum enrichment; Suppl, Supplementation; *T*_max_, time to maximum enrichment.

^3^GLIMMIX F test: Interaction is BiW × Suppl.

^a,b^Different from NBW piglets within Suppl group (*P* ≤ 0.05).

^c,d^Different from W supplemented piglets within BiW group (*P* ≤ 0.05).

## Discussion

The small intestine of LBW piglets has been reported to have a reduced absorptive surface area and compromised gut barrier function compared with NBW piglets (Everaert et al., 2017). These differences have been associated with impaired nutrient uptake, increased permeability, and a heightened risk of morbidity and mortality (Everaert et al., 2017). Supplemental Gln has been shown to improve enterocyte function, maintain gut integrity, and prevent the entry of pathogenic bacteria into the bloodstream in postweaning pigs (Blachier et al., 2007), and we have previously reported increased BW and jejunal villus height of suckling LBW piglets compared with LBW piglets supplemented with Ala (Li et al., 2022; Schregel et al., 2022). However, how Gln supports these improvements is unknown. Glutamine followed by Glc are preferred energy sources of intestinal enterocytes, where the majority of Gln carbon absorbed by the small intestine is metabolized in the Krebs cycle to provide energy, and released as CO_2_, while the remaining Gln can be incorporated into tissue protein, or used for the synthesis of AA such as Asp, Arg, Pro, ornithine and Cit, as well as for gluconeogenesis (Cruzat et al., 2018). The objectives of this study were to determine if Gln supplementation improves the BW, affects the intestinal permeability/absorptive capacity in suckling LBW and NBW pigs differently, and traces the metabolic fate of glutamine and glucose carbons using ^13^C_5_-Gln and ^13^C_6_-Glc tracers.

### Growth performance of low birthweight piglets supplemented with glutamine

Piglets with a low BiW reach slaughter weight later compared with their normal-weight littermates (Rehfeldt and Kuhn, 2006; Beaulieu et al., 2010), and studies have shown that Gln supplementation can improve the weight gain of LBW suckling (Haynes et al., 2009; Li et al., 2022; Schregel et al., 2022) and weaning piglets (Wu et al., 1996; Wang et al., 2015, 2022). In the present study, we observed that oral Gln supplementation did not improve BW in LBW piglets, contrary to previous studies conducted in suckling piglets by our group (Li et al., 2022; Schregel et al., 2022). The observation that LBW-Gln piglets had a lower BW than LBW-W prior to the catheter implantation was not observed in the NBW piglets, suggesting a mechanism unique to LBW piglets during this period. It could be assumed that differences in study design (higher litter size and parity) compared with the previous studies (Li et al., 2022; Schregel et al., 2022) might be a factor, but this would affect all piglets, regardless of BiW or supplementation. Another possible explanation is that LBW-Gln piglets utilize the extra Gln to prioritize intestinal over whole-body growth. However, in the current study, we did not find any difference in plasma Gln or Cit concentrations between LBW-Gln and LBW-W piglets, suggesting that there was no difference in Gln metabolism by the small intestine and that small intestine mass, as measured by plasma Cit concentrations, did not differ. This could indicate that the additional Gln was largely oxidized in the first pass and was not used for protein synthesis in the intestine. Furthermore, in another cohort of piglets, no difference in the weight or length of the duodenum and small intestine was observed between LBW-Gln and LBW-W piglets (manuscript in preparation). Thus, it is not clear, from the data reported in this study why Gln supplementation negatively affected BW in LBW piglets only, which may require further investigation.

### Glutamine metabolism

Under normal dietary conditions, approximately two thirds of dietary Gln taken up by the intestine is used for energy generation via the Krebs cycle (Stoll and Burrin, 2006; Cruzat et al., 2018). The five carbons from Gln enter the Krebs cycle as α-ketoglutarate, where two molecules of CO_2_ in one turn of the cycle are released (Newsholme and Carrie, 1994; Arnold and Finley, 2023). In this study, we administered an oral bolus of ^13^C_5_-Gln together with unlabeled Gln, to assess the metabolic fate of Gln carbons during our supplementation protocol. We observed that NBW piglets supplemented with Gln had a lower enrichment of ^13^C_3_-Glc in plasma at 30 and 60 min and a lower recovery of ^13^CO_2_ in RBC, while *T*_max_ of RBC ^13^CO_2_ and ^13^C_3_-Glc compared with LBW-Gln and NBW-W piglets were higher. These results suggest that, within the first hour postsupplementation, NBW-Gln piglets were absorbing and/or metabolizing Gln slower through the Krebs cycle to ^13^CO_2_ and ^13^C_3_-Glc than LBW-Gln and NBW-W. However, this did not affect the overall metabolism of ^13^C_5_-Gln to CO_2_ or ^13^C_3_-Glc, as the AUC of ^13^CO_2_ and ^13^C_3_-Glc was not different when NBW-Gln piglets were compared with LBW-Gln and NBW-W. However, it cannot be excluded that in the course of the tracer study, Gln uptake during suckling or endogenous Gln synthesis was greater in NBW piglets, and thus, the dilution of the ^13^C_5_-Gln tracer was greater than in the other groups. The ^13^C_3_-Glc enrichment, as well as ^13^C_5_-Gln to ^13^C_3_-Glc conversion, an indicator of Gln carbon used for gluconeogenesis, and ^13^C_5_-Gln Ra were not different between the groups. These results may indicate that the metabolism of ^13^C_5_-Gln differs only in the kinetics but not in magnitude among LBW and NBW piglets with or without Gln supplementation. Interestingly, the ^13^CO_2_ REC of ^13^C_5_-Gln, a proxy of whole-body Gln oxidation, was higher in LBW than NBW piglets, suggesting LBW piglets, regardless of supplementation, oxidize more Gln.

While the majority of Gln taken up by the small intestine is used for energy generation, it can also be used for the production of other AA via transamination such as Asp, Ala, Pro, and Cit (Stoll and Burrin, 2006; Wu et al., 2011; Blachier et al., 2013). We measured the plasma concentrations of Gln and eight AA linked to Gln metabolism to obtain additional information about AA metabolism. The plasma concentration of Gln peaked at 30 min for all groups and confirms results from a companion study in which 1 g/kg BW Gln was also supplemented and showed that plasma Gln peaks at 45 min (Li et al., 2022). The Gln peak was lower in Gln-supplemented piglets; however, the Gln concentration over the entire experimental period was lower in LBW compared with NBW piglets, probably indicating a higher glutaminase activity in LBW piglets. The deamination of Gln to Glu by glutaminase releases ammonia that is transported to the liver to be detoxified by the production of urea (Wu, 1995), and here, we report that during the Gln tracer test, LBW piglets had higher levels of plasma Glu and urea than NBW littermates. The Glu result should be interpreted with caution as there were differences between the experimental groups prior to bolus administration, with higher plasma Glu concentrations in LBW-W compared with LBW-Gln and NBW-W piglets. When Glu is converted into α-ketoglutarate, this can result in the production of Asp or Ala (Yoo et al., 2020), and we show higher plasma Asp but not Ala in LBW than NBW littermates during the test, suggesting that LBW piglets preferentially metabolized Gln to Asp and α-ketoglutarate.

The piglets were only separated from the sow 1 h prior to bolus administration to avoid starvation. Since piglets suckle the sow about once per h (Skok et al., 2007), a 1 h milk withdrawal is interpreted as an “acute milk withdrawal effect” on the plasma AA profile in response to this separation. This effect has been previously reported in 5 to 7 d piglets, where the plasma concentrations of 19 of the 20 proteinogenic AA and Cit decreased 2 h postfasting (Bertolo et al., 2000). It is therefore plausible that there was a slight fasting effect on the plasma AA profile. However, why this resulted in differences between the experimental groups remains to be investigated. It should be noted that plasma lactate concentrations have been used as a marker for fasting (Freminet et al., 1975), and in this study, the prebolus plasma lactate concentrations were between 5.5 and 7.4 mM, which is similar to blood lactate values of neonatal piglets with low colostrum intake (Quesnel et al., 2023). Postbolus, the plasma lactate concentrations decreased significantly compared with the prebolus (−15 min) period to under 2 mM, potentially due to being able to suckle once again. Finally, the differences in AA concentrations observed prebolus were present during most of the postbolus sampling time points, indicating that the bolus has no immediate effect on the levels of these plasma AAs. Another of the AA whose plasma concentration was higher prior to bolus administration was Cit, which was higher in NBW-Gln than NBW-W. However, results also show that during the test Gln supplemented piglets had higher plasma Cit concentrations than control piglets, likely due to the synthesis of Cit from Gln (Wu et al., 1994b). It is also noteworthy that Cit is primarily synthesized in the small intestine (Wu et al., 1994b), and it is considered a biomarker of intestinal mass (Lansing et al., 2019).

### Glucose metabolism, and intestinal absorptive capacity, malabsorption, and barrier function

Whilst glucose is the second preferred energy substrate for enterocytes of the small intestine, behind glutamine (Wu et al., 1995; Roth, 2008), the primary role of the small intestine is to transport dietary glucose into the circulation, where it is taken up by the liver and other organs and metabolized (Maggs et al., 2008). Its absorption from the lumen occurs in the small intestine via the sodium-glucose linked transporter 1, whose mRNA abundance in the jejunum was increased in weaned piglets fed 0.30 or 0.45% alanyl-glutamine for 21 d (Zou et al., 2019), suggesting that Glc uptake may be increased in piglets fed supplemental Gln. To this end, we administered an oral bolus containing ^13^C_6_-Glc and unlabeled-Glc, to assess the metabolic fate of Glc carbons. The plasma ^13^C_6_-Glc enrichment within the first 30 min after the oral bolus was higher in LBW piglets than in their NBW littermates. However, the plasma enrichment of ^13^C_6_-Glc at 1 h postbolus was higher only in LBW-W than NBW-W. The enrichment of ^13^C_6_-Glc can be affected by milk intake where the released glucose from lactose in milk dilutes the tracer, lowering enrichment values, which could suggest higher milk intake in NBW-W than LBW-W pigs.

Results showed also that at 150 and 180 min after the ^13^C_6_-Glc bolus, enrichment of ^13^CO_2_ in RBC was higher in NBW-Gln than NBW-W piglets and was associated with higher *E*_max_, *T*_max_, and AUC in NBW-Gln than NBW-W. These results suggest that NBW piglets supplemented with Gln oxidize more Glc to CO_2_ than those supplemented with water, and faster.

The ^13^C_6_-Glc/ Glc bolus also contained Xyl to assess small intestine absorptive capacity. In pigs, it is proposed that only a small amount of Xyl is absorbed via active transporters as described for glucose, with the majority of absorption expected to occur through diffusion (Huntley and Patience, 2018; Uken et al., 2021). Once taken up, Xyl is only marginally metabolized, and thus, a low blood level after Xyl administration is an indication of malabsorption by the intestine. The Xyl test has been performed in pre- and postweaning piglets (Miller et al., 1984; Hampson and Kidder, 1986; Hampson and Smith, 1986; Kelly et al., 1990), and the method involves taking a single blood sample 1 h after Xyl administration and any differences in Xyl concentrations are associated to differences in intestinal absorptive capacity (Miller et al., 1984; Hampson and Smith, 1986). In the present study, we collected blood samples over a 5 h period and assessed changes in plasma Xyl concentrations over this period. At 1 h postbolus, plasma Xyl concentrations were higher and *T*_max_ lower in LBW-Gln than in NBW-Gln, suggesting that absorption capacity is higher and absorption faster in LBW-Gln piglets compared with their NBW littermates. Studies in pigs and poultry indicate that Xyl is transported via similar sodium-dependent transporters as Glc (Huntley and Patience, 2018; Goutchtat, 2023). Therefore, it is plausible that LBW piglets had enhanced intestinal absorptive capacity, potentially through the upregulation of Glc / Xyl transporter gene expression.

Lower mannitol concentrations have been observed at 4 d postweaning, which correlated with reduced piglet growth, and was interpreted as mannitol and nutrient malabsorption (Berkeveld et al., 2008). The authors of another study reported a high correlation between jejunal villous height and the plasma mannitol concentration and concluded that mannitol levels are an indicator of small intestinal surface area (Turpin et al., 2017). We observed a higher plasma mannitol concentration in LBW piglets and LBW-Gln piglets at 30- and 60-min postbolus, respectively. Since mannitol is absorbed by transcellular passive and active routes, our results suggest a larger absorptive area in LBW piglets, which confirms our findings with the Xyl test. The fact that plasma lactulose was at or below limits of quantification in all piglet groups may indicate that the intestinal barrier function was not compromised in these animals. Our results seem to be in contrast to earlier reports concluding that in very young IUGR piglets, the absorptive area was smaller because of reduced intestinal villus height and crypt depth, and neonatal IUGR piglets show a higher transcellular permeability (Everaert et al., 2017). However, the 14 to 16 d old LBW piglets investigated in the present study are more mature than neonatal IUGR piglets in their first day of life and are therefore not comparable.

The lower plasma urea concentrations observed during the ^13^C_6_-Glc/unlabeled Glc/Xyl test period in Gln compared with W supplemented pigs suggests that the urea metabolism is affected by the bolus, but the data available from this study do not allow this to be explained.

### Comparison of glutamine and glucose rate of appearance and oxidation

Plasma Ra of ^13^C_5_-Gln compared with ^13^C_6_-Glc was lower in all experimental groups, which may indicate that enteral glutamine is taken up primarily in the small intestine (Stoll et al., 1998), associated to a lower appearance in the circulation. Although Glc is also an important fuel for the enterocytes of suckling piglets, Gln still represents the major oxidative energy substrate for enterocytes, as it is oxidized about eight times faster than in enterocytes isolated from weaned pigs (Darcy-Vrillon et al., 1994). Others reported that glucose oxidation in intestinal tissue slices is 100 times lower than the conversion of Glc to lactate (Girard et al., 1992), and only 15% of the total CO_2_ production in the portal drained viscera of piglets was from enteral glucose, but 36% from glutamate (presumably derived from Gln), respectively (Stoll et al., 1999). This is in agreement with reports that glucose supplied by the milk accounts for only 20% to 50% of glucose requirements and this emphasizes the importance of gluconeogenesis in suckling piglets (Girard et al., 1992). In the present study, we also observed a lower overall oxidation of Glc (^13^C REC) compared with Gln in all experimental groups, which is consistent with the metabolic strategy during suckling, namely the sparing of Glc for organs with an absolute glucose requirement (Girard et al., 1992). This also agrees with the higher Glc plasma Ra in our study, irrespective of BiW and supplementation. However, our in vivo results do not support the results of in vitro studies (Kight and Fleming, 1995; Quan et al., 1998) that glutamine reduces the oxidation of glucose. The comparison of LBW with NBW littermates resulted in a higher oxidation of Gln and Glc of LBW piglets with no effect of Gln supplementation. However, neonatal piglet survival depends on their ability to utilize milk fat as major fuel but show a low hepatic capacity to oxidize fatty acids and ketone bodies likely associated to a low carnitine palmitoyltransferase activity (Lin et al., 2007). This could indicate that the possibly less mature LBW piglets are more dependent on the utilization of glutamine and glucose for energy production, while the NBW piglets may transition more quickly to a lipid-based energy supply.

## Conclusions

In the present study, we observed that oral Gln supplementation was associated with a decrease in LBW piglet BW compared with water supplemented LBW piglets, contradicting results obtained in a previous study. Although differences in Gln and Glc metabolism were observed between the experimental groups, no consistent effect of Gln supplementation was seen. The absence of a consistent effect of Gln supplementation on Gln and Glc metabolism may be associated with the effects of the surgery, which negatively affected postsurgery BW gain. Comparison of the Gln and Glc Ra results showed that Gln has a lower Ra than Glc, whereas Glc oxidation (^13^C REC) was lower than that of Gln, which is most likely due to Gln being preferred by the small intestine as an energy source and glucose having to be spared during suckling. This confirms earlier findings that Gln is more important as a fuel for the intestine than Glc. It was striking that the oxidation of Gln and Glc was higher in LBW than in NBW piglets, suggesting a lower maturity of LBW compared with NBW piglets. Lastly, the Xyl test indicates that LBW piglets appear to have higher absorptive capacity in the small intestine regardless of supplementation.

## Supplementary Material

skaf201_suppl_Supplementary_Materials

## Data Availability

The datasets generated during and/or analyzed during the current study have been uploaded to zenodo and available at the following DOI: 10.5281/zenodo.13911423.

## Abbreviations

^13^C REC: ^13^C recovery
AA: amino acid
ABC: abdominal circumference
ADG: average daily gain
Ala: alanine
APE: atom percent excess
Arg: arginine
Asn: asparagine
Asp: aspartate
AUC: area under the curve
BiW: birthweight
BMI: body mass index
BW: bodyweight
Cit: citrulline
CP: crude protein
CRL: crown-rump length
EE: ether extract
E_max_: maximum enrichment value
FAA: free amino acids
FBN: Research Institute for Farm Animal Biology
Glc: glucose
Gln: glutamine
Glu: glutamate
IUGR: intrauterine growth restriction
LBW: low birthweight
Lac: lactulose
Man: mannitol
ME: metabolizable energy
MPE: molar percent excess
NBW: normal birthweight
PI: ponderal index
Pro: proline
Ra: rate of appearance
RBC: red blood cell
RT: rectal temperature
Ser: serine
T_max_: time to reach maximum enrichment
W: water
Xyl: xylose
