# Supplementary material for: Effect of oral glutamine supplementation on growth and glutamine and glucose metabolism in suckling piglets
Source: J Anim Sci. 2025 Jun 18;103:skaf201. doi: 10.1093/jas/skaf201 (PMC12267151; doi:10.1093/jas/skaf201)

Effect of oral glutamine supplementation on growth and glutamine and glucose metabolism in suckling piglets ^1^

Quentin Leon Sciascia,^*^ Daria De Leonardis,^*^ Solvig Görs,^*^ Andreas Vernunft,^*^ Anja Eggert,^*^ Jürgen Zentek,^†^ and Cornelia C. Metges^*,2^

^*^ Research Institute for Farm Animal Biology (FBN), 18196 Dummerstorf, Germany.

^†^ Freie Universität Berlin, Department of Veterinary Medicine, Institute of Animal Nutrition, 14163 Berlin, Germany

^1^ This work is an outcome of the MONOGUTHEALTH project, which has received funding from the European Union’s Horizon 2020 research and innovation programme under the Marie Skłodowska-Curie Innovative Training Networks grant agreement No 955374.

^2^ Corresponding author: [metges@fbn-dummerstorf.de](mailto:metges@fbn-dummerstorf.de)

“Online Supplementary Material”

**Supplementary Table 1.** Morphometric measures and average daily gain of low (LBW) and normal (NBW) birthweight (BiW) male piglets supplemented with glutamine (Gln; 1 g/kg bodyweight (BW) per d) or water (W), from 2 to 15 d of age.

| Parameters^1^ | Age^2^ | Gln | | | |  | W | | | |  | *P*-value^3^ | | | |
| --- | --- | --- | --- | --- | --- | --- | --- | --- | --- | --- | --- | --- | --- | --- | --- |
|  |  | LBW | SEM | NBW | SEM |  | LBW | SEM | NBW | SEM |  | BiW | Age | Supplementation | Interaction |
| Abdominal circumference, cm | 1 | 22.0^b^ | 0.54 | 25.7^a^ | 0.60 |  | 22.4^b^ | 0.56 | 26.4^a^ | 0.56 |  | *<0.001* | *<0.001* | 0.003 | 0.87 |
|  | 2 | 23.0^b^ | 0.54 | 25.5^a^ | 0.54 |  | 23.8^b^ | 0.66 | 26.5^a^ | 0.56 |  |  |  |  |  |
|  | 8 | 28.1^b,d^ | 0.62 | 32.2^a,d^ | 0.57 |  | 30.4^b,c^ | 0.58 | 33.8^a,c^ | 0.63 |  |  |  |  |  |
| Crown rump length, cm | 1 | 24.2^b^ | 0.51 | 26.8^a^ | 0.51 |  | 23.0^b^ | 0.54 | 27.0^a^ | 0.54 |  | *<0.001* | *<0.001* | 0.50 | 0.47 |
|  | 2 | 24.8^b^ | 0.53 | 27.5^a^ | 0.51 |  | 23.8^b^ | 0.61 | 26.9^a^ | 0.54 |  |  |  |  |  |
|  | 8 | 28.8^b^ | 0.55 | 32.8^a^ | 0.55 |  | 29.4^b^ | 0.64 | 33.1^a^ | 0.56 |  |  |  |  |  |
| Body mass index, kg/m^2^ | 1 | 18.2^b^ | 0.81 | 21.6^a^ | 0.81 |  | 19.5 | 0.85 | 21.9 | 0.98 |  | *<0.001* | *<0.001* | 0.05 | 0.88 |
|  | 2 | 17.1^b^ | 0.84 | 20.5^a^ | 0.84 |  | 19.2^b^ | 0.85 | 22.6^a^ | 0.85 |  |  |  |  |  |
|  | 8 | 23.9 | 0.88 | 26.1 | 0.93 |  | 23.9^b^ | 0.98 | 27.0^a^ | 0.89 |  |  |  |  |  |
| Ponderal index, kg/m^3^ | 1 | 76.5 | 4.29 | 81.7 | 4.29 |  | 81.7 | 4.71 | 81.4 | 4.91 |  | 0.50 | 0.32 | 0.16 | 0.87 |
|  | 2 | 69.3 | 4.46 | 74.2 | 4.46 |  | 80.5 | 4.51 | 84.6 | 4.51 |  |  |  |  |  |
|  | 8 | 83.1 | 4.66 | 79.3 | 4.89 |  | 81.5 | 5.2 | 82.1 | 4.72 |  |  |  |  |  |
| Average daily gain, g | 1 - 16 | 133^b^ | 8.54 | 211^a^ | 8.81 |  | 144^b^ | 8.80 | 190^a^ | 8.80 |  | *<0.001* | - | 0.56 | 0.02 |
| Pre-surgery | 1 - 12 | 158^b^ | 12.6 | 222^a^ | 12.6 |  | 180 | 13.4 | 206 | 13.4 |  | *<0.001* | - | 0.83 | 0.09 |
| Post-surgery | 13 - 16 | 25.2 | 9.22 | 39.3 | 8.59 |  | 19.8^b^ | 8.97 | 42.4^a^ | 8.78 |  | 0.002 | - | 0.83 | 0.42 |

^1^Values are least-square means ± SEM, LBW-Gln; n = 8-12, NBW-Gln; n = 9-12, LBW-W; n = 7-11; NBW-W, n = 8-11 animals per group.

^2^Age: days, day 1, day of birth.

^3^GLIMMIX F test: Interaction for abdominal circumference, crown rump length, body mass index and ponderal index is BiW × Supplementation × Age, Interaction for Average daily gain, pre- and post-surgery is BiW × Supplementation.

^a,b^Different from NBW piglets within Supplementation group (*P* ≤ 0.05).

^c,d^Different from W supplemented piglets within BiW group (*P* ≤ 0.05).

**Supplementary Table 2**. Average free alanine, arginine, asparagine, proline, and serine concentrations in plasma after a bolus dose of glutamine (Gln; 0.33 g/kg bodyweight (BW)) plus ^13^C_5_-Gln (10 mg/kg BW) at 14 d of age in low (LBW) and normal (NBW) birthweight (BiW) male piglets supplemented with Gln (1 g/kg BW per d) or water (W), from 2 to 15 d of age

| Amino acids^1^ | Gln | | | |  | W | | | |  | *P*-value^2^ | |
| --- | --- | --- | --- | --- | --- | --- | --- | --- | --- | --- | --- | --- |
| µmol/L | LBW | SEM | NBW | SEM |  | LBW | SEM | NBW | SEM |  | Time | Interaction |
| Alanine | 355 | 65.7 | 401 | 65.3 |  | 408 | 66.7 | 452 | 66.1 |  | 0.03 | 0.30 |
| Arginine | 119 | 21.8 | 117 | 21.7 |  | 107 | 22.1 | 115 | 21.9 |  | 0.41 | 0.53 |
| Asparagine | 35.6 | 6.54 | 37.6 | 6.49 |  | 36.7 | 6.53 | 34.2 | 6.51 |  | 0.09 | 0.31 |
| Proline | 339 | 54.6 | 362 | 53.3 |  | 367 | 55.3 | 373 | 54.1 |  | *<0.001* | 0.01 |
| Serine | 142 | 18.0 | 134 | 17.8 |  | 152 | 18.1 | 146 | 17.9 |  | *< 0.001* | 0.41 |

^1^Values are least-square means ± SEM calculated from time 0 to 300 min after the tracer bolus, LBW-Gln; n = 5-10, NBW-Gln; n = 5-11, LBW-W; n = 6-8; NBW-W, n = 6-11 animals per group.

^2^GLIMMIX F test: Interaction is BiW × time. The factors BiW, Supplementation, Supplementation × Time and BiW × Supplementation × Time were not significant (*P* > 0.1).

**Supplementary Table 3.** Glucose, lactate and urea concentrations in plasma after a bolus dose of glutamine (Gln; 0.33 g/kg bodyweight (BW)) plus ^13^C_5_-Gln (10 mg/kg BW) at 14 d of age in low (LBW) and normal (NBW) birthweight (BiW) male piglets supplemented with Gln (1 g/kg BW per d) or water (W), from 2 to 15 d of age

| Parameters^1^ | Time^2^ | Gln | | | |  | W | | | |  | *P*-value^3^ | | |
| --- | --- | --- | --- | --- | --- | --- | --- | --- | --- | --- | --- | --- | --- | --- |
|  |  | LBW | SEM | NBW | SEM |  | LBW | SEM | NBW | SEM |  | BiW | Time | Interaction |
| Glucose | -15 | 4.92^c^ | 2.06 | 4.73 | 2.00 |  | 5.88^a,d^ | 3.12 | 5.04^b^ | 1.45 |  | 0.32 | 0.01 | 0.11 |
| mmol/L | 30 | 5.05 | 1.20 | 4.78 | 1.84 |  | 5.02 | 0.74 | 4.84 | 1.45 |  |  |  |  |
|  | 60 | 5.07 | 1.14 | 5.01 | 0.99 |  | 5.24 | 1.88 | 5.02 | 1.69 |  |  |  |  |
|  | 90 | 5.01 | 0.83 | 4.83 | 1.31 |  | 5.50 | 1.16 | 5.34 | 0.93 |  |  |  |  |
|  | 120 | 4.48 | 0.60 | 5.10 | 1.33 |  | 5.44 | 0.75 | 5.02 | 0.99 |  |  |  |  |
|  | 150 | 5.31 | 2.27 | 5.28 | 1.21 |  | 5.75 | 0.82 | 5.28 | 1.56 |  |  |  |  |
|  | 180 | 4.94 | 1.22 | 5.09 | 1.01 |  | 5.41 | 1.05 | 4.84 | 1.11 |  |  |  |  |
|  | 210 | 4.63 | 1.79 | 4.83 | 1.50 |  | 5.39 | 1.43 | 5.30 | 1.58 |  |  |  |  |
|  | 240 | 5.06 | 1.34 | 5.03 | 0.68 |  | 5.07 | 1.30 | 4.96 | 1.12 |  |  |  |  |
|  | 300 | 5.48 | 0.97 | 5.30 | 1.03 |  | 5.57 | 1.52 | 5.42 | 1.34 |  |  |  |  |
| Lactate | -15 | 5.81 | 0.50 | 5.47 | 0.68 |  | 6.63 | 1.00 | 7.42 | 1.00 |  | 0.85 | *<0.001* | 0.54 |
| mmol/L | 30 | 2.63 | 0.63 | 1.67 | 0.17 |  | 2.65 | 0.41 | 2.62 | 0.42 |  |  |  |  |
|  | 60 | 1.57 | 0.30 | 1.56 | 0.20 |  | 1.92 | 0.41 | 1.90 | 0.39 |  |  |  |  |
|  | 90 | 1.61 | 0.37 | 1.42 | 0.15 |  | 1.96 | 0.26 | 1.91 | 0.39 |  |  |  |  |
|  | 120 | 1.74 | 0.31 | 1.63 | 0.24 |  | 2.31 | 0.46 | 2.29 | 0.45 |  |  |  |  |
|  | 150 | 1.59 | 0.35 | 1.61 | 0.25 |  | 2.00 | 0.32 | 1.64 | 0.20 |  |  |  |  |
|  | 180 | 1.82 | 0.35 | 1.67 | 0.19 |  | 1.99 | 0.29 | 2.04 | 0.48 |  |  |  |  |
|  | 210 | 1.51 | 0.35 | 1.65 | 0.29 |  | 1.98 | 0.53 | 1.91 | 0.36 |  |  |  |  |
|  | 240 | 1.47 | 0.34 | 1.52 | 0.30 |  | 2.13 | 0.55 | 1.72 | 0.28 |  |  |  |  |
|  | 300 | 1.47 | 0.13 | 1.97 | 0.20 |  | 1.85 | 0.36 | 1.54 | 0.25 |  |  |  |  |
| Urea | -15 | 3.50 | 0.42 | 2.73 | 0.22 |  | 3.10 | 0.55 | 2.31 | 0.23 |  | 0.02 | *<0.001* | *<0.001* |
| mmol/L | 30 | 3.37 | 0.56 | 2.50^c^ | 0.21 |  | 2.55^a^ | 0.48 | 1.87^b,d^ | 0.19 |  |  |  |  |
|  | 60 | 3.54 | 0.54 | 3.27 | 0.77 |  | 2.69 | 0.44 | 2.13 | 0.17 |  |  |  |  |
|  | 90 | 3.61 | 0.52 | 3.17 | 0.60 |  | 2.89 | 0.43 | 2.30 | 0.31 |  |  |  |  |
|  | 120 | 3.67 | 0.49 | 3.53 | 0.84 |  | 3.33 | 0.60 | 2.63 | 0.31 |  |  |  |  |
|  | 150 | 4.05 | 0.46 | 3.52 | 0.81 |  | 3.44 | 0.56 | 2.64 | 0.37 |  |  |  |  |
|  | 180 | 4.10 | 0.46 | 3.59 | 0.79 |  | 3.35 | 0.45 | 2.53 | 0.23 |  |  |  |  |
|  | 210 | 3.52 | 0.31 | 3.47 | 0.81 |  | 3.22 | 0.47 | 2.62 | 0.33 |  |  |  |  |
|  | 240 | 4.07 | 0.56 | 2.82 | 0.35 |  | 3.20 | 0.39 | 2.49 | 0.39 |  |  |  |  |
|  | 300 | 3.91 | 0.45 | 2.81 | 0.21 |  | 3.32 | 0.44 | 2.71 | 0.30 |  |  |  |  |

^1^Values are observed means ± SEM, LBW-Gln; n = 8-12, NBW-Gln; n = 7-12, LBW-W; n = 10; NBW-W, n = 9-11 animals per group.

^2^Time: min.

^3^GLIMMIX F test: Interaction is Supplementation × Time. The factors Supplementation, BiW × Time and BiW × Supplementation × Time were not significant (*P* > 0.1).

^a,b^Different from NBW piglets within Supplementation group (*P* ≤ 0.05).

^c,d^Different from W supplemented piglets within BiW group (*P* ≤ 0.05).

**Supplementary Table 4.** Glucose, lactate, and urea concentrations in plasma after a bolus dose of glucose (Glc; 0.4 g/kg bodyweight (BW)) plus ^13^C_6_-Glc (10 mg/kg BW) and xylose (0.4 g/kg BW) at 16 d of age in low (LBW) and normal (NBW) birthweight (BiW) male piglets supplemented with glutamine (Gln; 1 g/kg BW per d) or water (W), from 2 to 15 d of age

| Parameters^1^ | Time^2^ | Gln | | | |  | W | | | |  | *P*-value^3^ | | |
| --- | --- | --- | --- | --- | --- | --- | --- | --- | --- | --- | --- | --- | --- | --- |
|  |  | LBW | SEM | NBW | SEM |  | LBW | SEM | NBW | SEM |  | Supplementation | Time | Interaction |
| Glucose | -15 | 3.84 | 0.56 | 3.81 | 0.55 |  | 3.48 | 0.58 | 3.26 | 0.56 |  | 0.46 | 0.020 | 0.20 |
| mmol/L | 30 | 3.88 | 0.56 | 3.91 | 0.55 |  | 4.01 | 0.58 | 3.72 | 0.56 |  |  |  |  |
|  | 60 | 3.95 | 0.56 | 3.90 | 0.55 |  | 3.68 | 0.58 | 3.52 | 0.56 |  |  |  |  |
|  | 90 | 3.94 | 0.56 | 3.91 | 0.55 |  | 3.61 | 0.58 | 3.53 | 0.56 |  |  |  |  |
|  | 120 | 3.54 | 0.56 | 3.54 | 0.55 |  | 3.57 | 0.58 | 3.18 | 0.57 |  |  |  |  |
|  | 150 | 3.55 | 0.56 | 4.15 | 0.55 |  | 3.54 | 0.58 | 3.41 | 0.56 |  |  |  |  |
|  | 180 | 3.57 | 0.56 | 3.68 | 0.55 |  | 3.72 | 0.58 | 3.49 | 0.56 |  |  |  |  |
|  | 210 | 3.93 | 0.56 | 3.92 | 0.55 |  | 3.65 | 0.58 | 3.58 | 0.56 |  |  |  |  |
|  | 240 | 3.49 | 0.57 | 3.89 | 0.55 |  | 3.81 | 0.58 | 3.85 | 0.56 |  |  |  |  |
|  | 300 | 4.03 | 0.56 | 3.66 | 0.55 |  | 3.86 | 0.58 | 3.69 | 0.56 |  |  |  |  |
| Lactate | -15 | 3.63^a,c^ | 0.20 | 2.47^b^ | 0.19 |  | 1.96^d^ | 0.28 | 1.97 | 0.22 |  | 0.06 | *<0.001* | *<0.001* |
| mmol/L | 30 | 1.58 | 0.20 | 1.45 | 0.20 |  | 1.87 | 0.22 | 1.95 | 0.21 |  |  |  |  |
|  | 60 | 1.15 | 0.21 | 0.98 | 0.22 |  | 1.17 | 0.24 | 1.07 | 0.21 |  |  |  |  |
|  | 90 | 1.56 | 0.24 | 1.08 | 0.21 |  | 1.10 | 0.24 | 0.82 | 0.21 |  |  |  |  |
|  | 120 | 1.50 | 0.21 | 1.28 | 0.19 |  | 0.99 | 0.23 | 0.89 | 0.21 |  |  |  |  |
|  | 150 | 1.27 | 0.23 | 1.25 | 0.20 |  | 1.08 | 0.22 | 0.91 | 0.24 |  |  |  |  |
|  | 180 | 1.51 | 0.23 | 1.16 | 0.20 |  | 1.06 | 0.22 | 0.94 | 0.22 |  |  |  |  |
|  | 210 | 1.15 | 0.24 | 0.96 | 0.22 |  | 0.84 | 0.28 | 1.10 | 0.23 |  |  |  |  |
|  | 240 | 1.09 | 0.23 | 1.38 | 0.19 |  | 0.86 | 0.28 | 1.38 | 0.20 |  |  |  |  |
|  | 300 | 1.44 | 0.21 | 1.24 | 0.20 |  | 1.15 | 0.23 | 1.07 | 0.21 |  |  |  |  |
| Urea | -15 | 2.71 | 0.30 | 2.75 | 0.29 |  | 2.32 | 0.33 | 2.26 | 0.30 |  | 0.01 | *<0.001* | 0.31 |
| mmol/L | 30 | 2.15 | 0.30 | 2.35^c^ | 0.29 |  | 2.12 | 0.33 | 1.62^d^ | 0.31 |  |  |  |  |
|  | 60 | 2.25 | 0.30 | 2.54^c^ | 0.29 |  | 1.96 | 0.33 | 1.60^d^ | 0.32 |  |  |  |  |
|  | 90 | 2.37 | 0.31 | 2.57^c^ | 0.29 |  | 1.86 | 0.33 | 1.63^d^ | 0.30 |  |  |  |  |
|  | 120 | 2.45 | 0.31 | 2.37^c^ | 0.29 |  | 1.92 | 0.33 | 1.63^d^ | 0.30 |  |  |  |  |
|  | 150 | 2.27 | 0.31 | 2.53^c^ | 0.29 |  | 1.78 | 0.33 | 1.47^d^ | 0.32 |  |  |  |  |
|  | 180 | 2.31 | 0.31 | 2.44^c^ | 0.29 |  | 1.88 | 0.33 | 1.52^d^ | 0.31 |  |  |  |  |
|  | 210 | 2.46 | 0.32 | 2.47^c^ | 0.29 |  | 1.89 | 0.33 | 1.63^d^ | 0.31 |  |  |  |  |
|  | 240 | 2.28 | 0.31 | 2.44^c^ | 0.29 |  | 1.76 | 0.32 | 1.71^d^ | 0.30 |  |  |  |  |
|  | 300 | 2.38 | 0.30 | 2.32^c^ | 0.29 |  | 1.82 | 0.33 | 1.55^d^ | 0.32 |  |  |  |  |

^1^Values are least-square means ± SEM, LBW-Gln; n = 6-11, NBW-Gln; n = 8-12, LBW-W; n = 5-9; NBW-W, n = 6-11 animals per group.

^2^Time: min.

^3^GLIMMIX F test: Interaction is Supplementation × Time. The factors BiW, BiW × Time and BiW × Supplementation × Time were not significant (*P* > 0.01).

^a,b^Different from NBW piglets within Supplementation group (*P* ≤ 0.05).

^c,d^Different from W supplemented piglets within BiW group (*P* ≤ 0.05).

**Supplementary Table 5.** Mannitol concentrations in plasma after a bolus dose of lactulose (0.75 g/kg BW) and mannitol (0.3 g/kg BW) at 15 d of age in low (LBW) and normal (NBW) birthweight (BiW) male piglets supplemented with glutamine (Gln; 1 g/kg bodyweight (BW)) or water (W), from 2 to 15 d of age

| Parameter^1^ | Time^2^ | Gln | | | |  | W | | | |  | *P*-value^3^ | |
| --- | --- | --- | --- | --- | --- | --- | --- | --- | --- | --- | --- | --- | --- |
|  |  | LBW | SEM | NBW | SEM |  | LBW | SEM | NBW | SEM |  | Time | BiW × Supplementation |
| Mannitol, mmol/L | -15 | 0.00 | 0.00 | 0.00 | 0.00 |  | 0.00 | 0.00 | 0.00 | 0.00 |  | *<0.001* | 0.90 |
|  | 30 | 0.24 | 0.09 | 0.19 | 0.09 |  | 0.18 | 0.09 | 0.23 | 0.09 |  |  |  |
|  | 60 | 0.50 | 0.09 | 0.46 | 0.09 |  | 0.45 | 0.09 | 0.42 | 0.09 |  |  |  |
|  | 90 | 0.84 | 0.09 | 0.70 | 0.09 |  | 0.66 | 0.09 | 0.67 | 0.09 |  |  |  |
|  | 120 | 0.96 | 0.09 | 0.79 | 0.09 |  | 0.79 | 0.09 | 0.79 | 0.09 |  |  |  |
|  | 150 | 1.02^a^ | 0.09 | 0.80^b^ | 0.09 |  | 0.83 | 0.09 | 0.80 | 0.09 |  |  |  |
|  | 180 | 0.86 | 0.09 | 0.79 | 0.09 |  | 0.75 | 0.09 | 0.74 | 0.09 |  |  |  |
|  | 210 | 0.75 | 0.09 | 0.69 | 0.09 |  | 0.65 | 0.09 | 0.67 | 0.09 |  |  |  |
|  | 240 | 0.62 | 0.09 | 0.60 | 0.09 |  | 0.51 | 0.09 | 0.53 | 0.09 |  |  |  |
|  | 300 | 0.40 | 0.09 | 0.39 | 0.100 |  | 0.37 | 0.100 | 0.39 | 0.09 |  |  |  |
| AUC, mmol/L × min | (0 – 300 min) | 197 | 20.2 | 183 | 21.0 |  | 164 | 21.6 | 165 | 21.6 |  | - | 0.68 |
| E_max_, mmol/L | - | 1.04 | 0.11 | 0.89 | 0.11 |  | 0.86 | 0.12 | 0.82 | 0.12 |  | - | 0.58 |
| T_max_, min | - | 139 | 9.68 | 150 | 9.68 |  | 150^a^ | 10.2 | 129^b^ | 10.2 |  | - | 0.01 |

^1^Values are least-square means ± SEM, LBW-Gln; n = 11, NBW-Gln; n = 7-11, LBW-W; n = 7-10; NBW-W, n = 9-10 animals per group.

^2^Time: min.

^3^GLIMMIX F test: Interaction is Supplementation × Time. The factors BiW, Supplementation, BiW × Time and BiW × Supplementation × Time were not significant (*P* > 0.1).

^a,b^Different from NBW piglets within Supplementation group (*P* ≤ 0.05).

**Supplementary Figure 1.** ^13^C enrichment in CO_2_ in breath compared to CO_2_ in red blood cells. (**A**) after a bolus dose of glutamine (Gln; 0.33 g/kg bodyweight (BW)) plus ^13^C_5_-Gln (10 mg/kg BW), (**B**) after a bolus dose of glucose (Glc; 0.4 g/kg bodyweight (BW)) plus ^13^C_6_-Glc (10 mg/kg BW) and xylose (0.4 g/kg BW) in piglets (14 - 16 d of age; n=4 piglets with 10 time points each for both tests in a pre-trial)


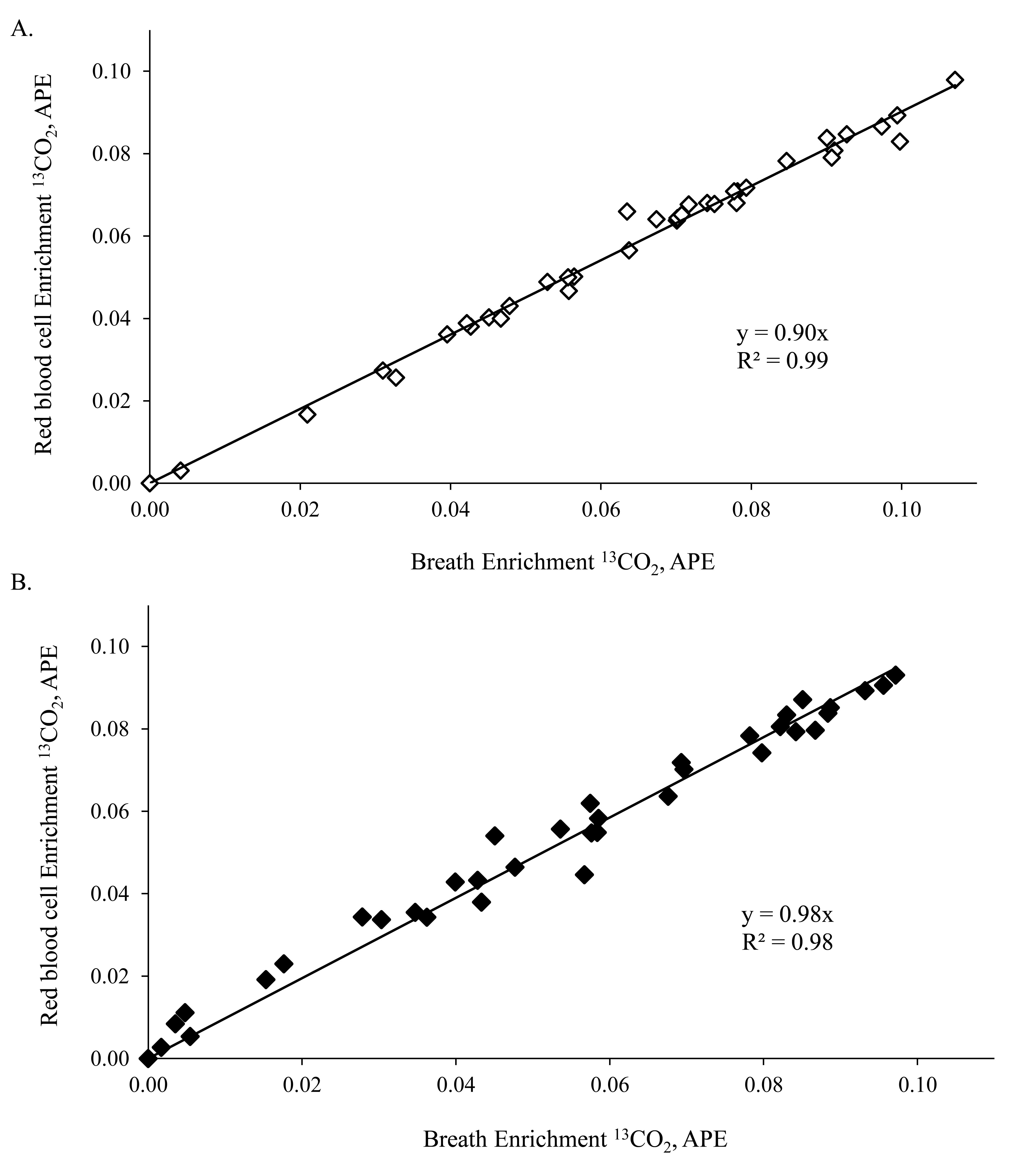

Supplement: skaf201_suppl_Supplementary_Materials [file skaf201_suppl_supplementary_materials.docx]
